# Development of Hydroxamate Derivatives Containing a Pyrazoline Moiety as APN Inhibitors to Overcome Angiogenesis

**DOI:** 10.3390/molecules27238339

**Published:** 2022-11-29

**Authors:** Yangyang Liu, Dongsheng Zhao, Chenghua Zhang, Hui Fang, Qingsitong Shen, Zhixian Wang, Jiangying Cao

**Affiliations:** School of Pharmacy, Shandong University of Traditional Chinese Medicine, Jinan 250355, China

**Keywords:** aminopeptidase N, CD13, inhibitors, antitumor agents, anti-angiogenesis

## Abstract

Aminopeptidase N (APN) was closely associated with cancer invasion, metastasis, and angiogenesis. Therefore, APN inhibitors have attracted more and more attention of scientists as antitumor agents. In the current study, we designed, synthesized, and evaluated one new series of pyrazoline-based hydroxamate derivatives as APN inhibitors. Moreover, the structure–activity relationships of those were discussed in detail. 2,6-Dichloro substituted compound **14o** with R_1_ = CH_3_, showed the best capacity for inhibiting APN with an IC_50_ value of 0.0062 ± 0.0004 μM, which was three orders of magnitude better than that of the positive control bestatin. Compound **14o** possessed both potent anti-proliferative activities against tumor cells and potent anti-angiogenic activity. At the same concentration of 50 μM, compound **14o** exhibited much better capacity for inhibiting the micro-vessel growth relative to bestatin in the rat thoracic aorta ring model. Additionally, the putative interactions of **14o** with the active site of APN are also discussed. The hydroxamate moiety chelated the zinc ion and formed four hydrogen bonds with His^297^, Glu^298^ and His^301^. Meanwhile, the terminal phenyl group and another phenyl group of **14o** interacted with S_2_′ and S_1_ pockets via hydrophobic effects, respectively.

## 1. Introduction

Aminopeptidase N (APN, CD13, EC 3.4.11.2), a type II transmembrane glycoprotein, is a zinc-dependent exopeptidase belonging to the M1 superfamily of the MA clan of peptidases [1]. APN is widely expressed on the surfaces of diverse cells, such as liver, renal, intestinal, fibroblast, endothelial, tumor cells and so on. Due to its multiple functions in the hydrolysis of peptides, signal transduction and animal coronavirus infection, APN is also called the “moonlighting enzyme” [2]. As an exopeptidase, APN preferentially cleaves the hydrophobic and basic amino acid residues from the N-terminus of biologically active peptides with broad substrate specificity [3]. For example, in the central nervous system, APN and neutral endopeptidase (NEP) cooperatively hydrolyzes enkephalin and endorphin to regulate pain [4]. Moreover, APN catalyzes the conversion of angiotensin III to angiotensin IV to regulate the renin–angiotensin system [5].

Ongoing interest is focused on APN due to its significant role in the metastasis and angiogenesis of tumors. Metastasis is a complex biological process involving cell migration, cell invasion and angiogenesis, which leads to more than 90% of cancer related deaths [6,7]. APN can metabolize the extracellular matrix (ECM) to promote the process of invasion and metastasis of tumor cells [8,9]. Moreover, expression of APN had been observed on the surfaces of angiogenic blood vessels rather than normal ones [10]. Impaired neovascularization was found in APN knockout mice [11]. Furthermore, it was reported that APN on the surfaces of both tumor cells and nonmalignant stromal cells cooperatively induced angiogenesis [12]. In addition, a strong correlation between up-regulated APN expression and poor prognosis of tumors has been certified through much research [13]. A recent study showed that APN was over-expressed in semi-quiescent liver cancer cells and considered as one of surface markers of liver cancer stem cells (CSCs), which mainly led to tumor recurrence [14]. All of this evidence supports the rationale that APN is an effective target for the chemotherapy of tumors.

**Figure 1:**
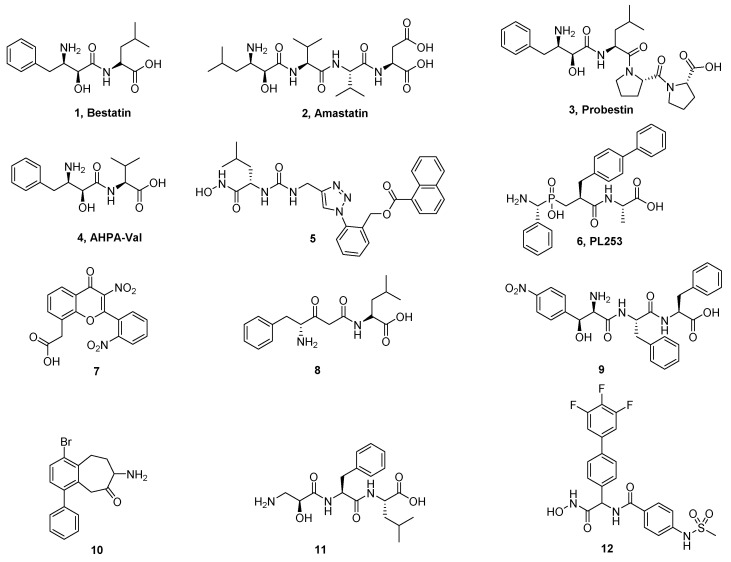
The structures of representative aminopeptidase N inhibitors (APNIs).

Structurally, APN is composed of 967 amino acids, contains a short intracellular tail, a transmembrane anchor, a small Ser-/Thr-rich extracellular stalk and a large ectodomain [15]. Domain II, one component of the ectodomain, contains one catalytic zinc ion and two characteristic motifs (HEXXHX18E zinc-binding motif and GXMEN substrate-recognition motif) from the M1 aminopeptidase family [16]. Around the zinc ion, there were three hydrophobic pockets S_1_, S_1_′ and S_2_′, which could bind the P_1_, P_1_′ and P_2_′ sites of the substrates, respectively. So far, many natural products and synthetic small molecules are reported to be APN inhibitors (APNIs). The natural APNIs are mostly peptides or peptidomimetics, such as bestatin **1**, amastatin **2**, probestin **3**, AHPA-Val **4**, and so on (Figure 1) [17,18]. The synthetic APNIs are structurally diverse, containing 1,2,3-triazol ureido peptidomimetics **5** [19], an aminophosphinic derivative **6** (PL253) [20], a flavone derivative **7** [21], a dicarbonyl derivative **8** [22], a chloramphenicol amine derivative **9** [23], an amino benzosuberone derivative **10** [24], a L-isoserine derivative **11** [25], an amide derivative **12** and so on (Figure 1) [26]. Among them, the dipeptide derivative bestatin is marketed as an immunomodulating agent for the adjuvant treatment of acute myeloid leukemia. Many studies have subsequently revealed that bestatin could prevent the invasion, metastasis and angiogenesis associated with tumors [27,28].

**Figure 2:**
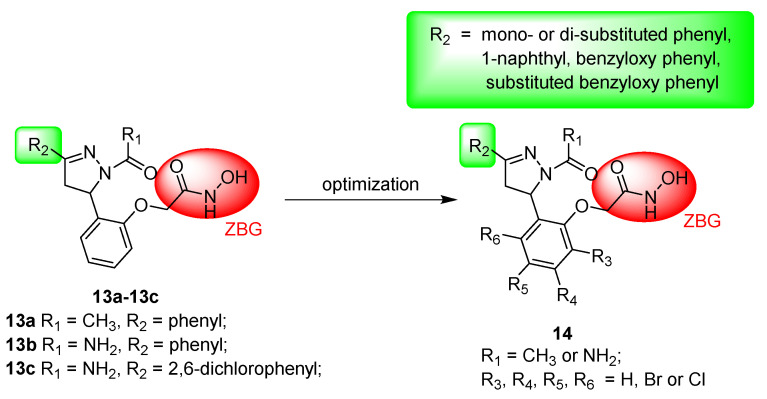
Design strategy of APNIs containing pyrazoline moiety.

The structure–activity relationships (SARs) of APNIs revealed that the potent APNIs possessed zinc binding groups (ZBGs) and hydrophobic groups interacting with at least one of three hydrophobic pockets (S_1_, S_1_′ and S_2_′) of APN. In our previous work, pyrazoline-based hydroxamate derivatives **13a** and **13b** showed the capacity of inhibiting APN (Figure 2) [29]. Subsequently, optimization focused on the terminal phenyl group of the diphenyl pyrazoline core gave compound **13c** with improved APN inhibitory activity relative to compounds **13a** and **13b** [30]. In this work, in order to extend the SARs of these series of compounds and find more potent APNIs, further optimization was focused on two phenyl groups of compounds **13a** and **13b**, leading to compound **14**. The introduction of hydrophobic substituents on the phenyl groups of compound **14** or the replacement of the terminal phenyl group with a naphthyl moiety may strengthen the hydrophobic interactions with APN. Interestingly, among all the newly synthesized compounds, compound **14o** presented the best APN inhibitory activity, being two orders of magnitude better than those of lead compounds **13a** and **13b**. 

## 2. Results and Discussion

### 2.1. Chemistry

**Scheme 1:**
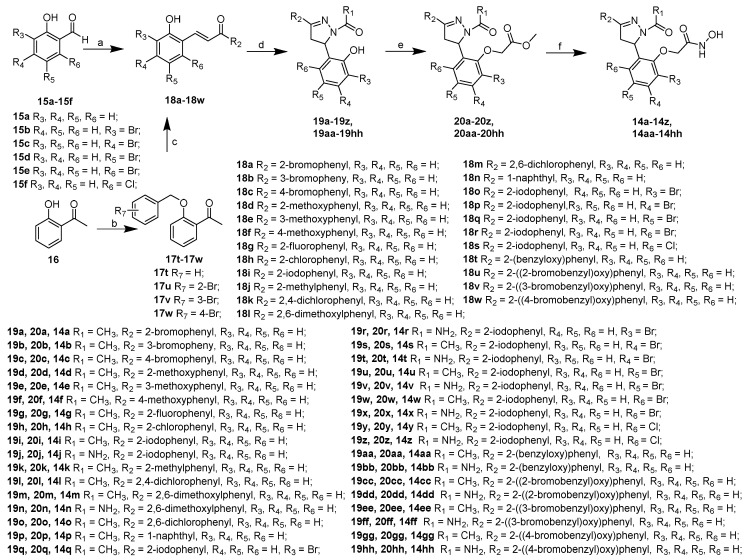
Reagents and conditions: (**a**) substituted acetophenone for **18a**–**18m, 18o**–**18s,** 1-(naphthalen-1-yl)ethan-1-one for **18n**, KOH, 80% EtOH, 25 °C, 48 h; (**b**) benzyl bromide or substituted benzyl bromide, NaH, DMF, 25 °C, 12 h; (**c**) for **18t**–**18w**, **15a**, KOH, 80% EtOH, 25 °C, 48 h; (**d**) R_1_ = CH_3_, acetic acid, 80% hydrazine hydrate, 130 °C, 5 h or R_1_ = NH_2_, semicarbazide hydrochloride, NaOH, EtOH, 78 °C, 5 h; (**e**) methyl bromoacetate, NaH, DMF, 25 °C, 12 h; (**f**) NH_2_OK, MeOH, 25 °C, 0.5 h.

The target compounds were synthesized following the procedures shown in Scheme 1. Firstly, benzaldehyde derivatives **15a**–**15f** reacted with the corresponding ketone to form the key chalcone intermediates **18a**–**18s** via the Claisen–Schmidt reaction in the presence of potassium hydroxide. Additionally, 2-hydroxyacetophenone **16** reacted with benzyl bromide or substituted benzyl bromide to give compounds **17t**–**17w**, which further reacted with 2-hydroxybenzaldehyde **15a** to give chalcone intermediates **18t**–**18w**, respectively, following the same procedure of compounds **18a**–**18s**. Subsequently, compounds **18a**–**18w** were cyclized with excess hydrazine hydrate or semicarbazide hydrochloride to give the pyrazoline derivatives **19a**–**19z** and **19aa**–**19hh**, which further underwent nucleophilic substitution reaction with methyl bromoacetate to yield compounds **20a**–**20z** and **20aa**–**20hh**, respectively. Ultimately, the freshly prepared methanolic solution of NH_2_OK transformed the methyl ester groups of **20a**–**20z** and **20aa**–**20hh** to hydroxamate groups to form the target compounds **14a**–**14z** and **14aa**–**14hh**, respectively.

### 2.2. Inhibitory Activities of Target Compounds against APN

All the synthesized pyrazoline derivatives were first evaluated for their APN inhibitory activities using bestatin and compounds **13a**–**13c** as the positive controls. The results are listed in Table 1. Similar to SARs previously reported, the substituents on the terminal phenyl group of the diphenyl pyrazoline moiety could significantly impact the inhibitory activities against APN [30]. Comparing mono-substituted compounds **14a**–**14f** with a methyl group as R_1_, it was easily found that compounds (**14a**, **14d**) with *ortho*-substituents showed better inhibitory potencies against APN than their counterparts with *meta*-substituents (**14b**, **14e**) or *para*-substituents (**14c**, **14f**), respectively. Moreover, among *ortho* mono-substituted compounds **14a**, **14d**, **14g**–**14i** and **14k**, compound **14i** containing an *ortho*-iodine substitution exhibited the best capacity of inhibiting APN, with an IC_50_ value of 0.015 ± 0.001 μM. The APN inhibitory order of *ortho* mono-substituted compounds with R_1_ = CH_3_, was **14i** (2-I) > **14h** (2-Cl) > **14a** (2-Br) > **14k** (2-CH_3_) > **14g** (2-F) > **14d** (2-OCH_3_). Among dual-substituted compounds (**14l**, **14m**, **14o**) with R_1_ = CH_3_, compound **14o** with 2,6-dichlorine substitution was two orders of magnitude better against APN than the analogues with 2,4-dichlorine (**14l**) or 2,6-dimethoxy (**14m**) substitution, suggesting that the substituents and substituted position on the terminal phenyl group significantly affected the inhibitory activities against APN. Moreover, *ortho* dual-substituted compounds showed improved APN inhibitory activities relative to the corresponding *ortho* mono-substituted analogs (**14m** vs. **14d**; **14o** vs. **14h**). In addition, the APN inhibitory activities of compounds **14j** and **14n**, with NH_2_ as R_1_, were inferior to those of counterparts **14i** and **14m**, with CH_3_ as R_1_, respectively. Replacement of the terminal phenyl group of compound **13a** with a naphthyl group led to compound **14p**, with comparable APN inhibitory activity to **13a**.

Moreover, in order to explore the APN inhibitory effects of substituents on the other phenyl group, compounds **14q**–**14z** were designed using **14i** and **14j** as lead compounds. Compound **14w**, with R_1_ = CH_3_ and R_6_ = Br, exhibited better APN inhibitory activity than the counterparts **14q** (R_3_ = Br), **14s** (R_4_ = Br) and **14u** (R_5_ = Br), respectively. The same inhibitory tendency was also found among compounds **14r**, **14t**, **14v** and **14x**, with R_1_ = NH_2_. Furthermore, compounds **14y** (IC_50_ = 0.023 ± 0.001 μM) and **14z** (IC_50_ = 0.070 ± 0.003 μM), with R_6_ = Cl, displayed comparable or improved APN inhibition relative to corresponding bromo-substituted compounds **14w** and **14x**, respectively. However, the APN inhibitory activities of compounds **14y** and **14z** were inferior to those of corresponding lead compounds **14i** and **14j**, respectively, indicating that the introduction of substituents on the other phenyl group was detrimental.

Furthermore, introduction of one large hydrophobic *ortho*-benzyloxyl group on the terminal phenyl group of compounds **13a** and **13b** led to compounds **14aa** and **14bb**, respectively, with one order of magnitude improved APN inhibitory activities. However, bromo-substitution of the benzyloxyl group of **14aa** and **14bb** led to decreased APN inhibition (**14cc**, **14ee**, **14gg** vs. **14aa**; **14dd**, **14ff**, **14hh** vs. **14bb**). Moreover, *meta* bromo-substituted compounds presented better APN inhibitory activities than the counterparts with *ortho* or *para* bromo-substitution (**14ee** vs. **14cc**, **14gg**; **14ff** vs. **14dd**, **14hh**). 

Generally, compounds with R_1_ = CH_3_, exhibited superior or inferior APN inhibitory activities to their counterparts with R_1_ = NH_2_, which depended on the substituents of the two phenyl groups of the diphenyl pyrazoline moiety. Interestingly, the APN inhibitory activities of compounds **14a**, **14h-14j**, **14o**, **14w**, **14y, 14aa**, **14bb** and **14ee** were better than those of lead compounds **13a-13c**, supporting our design strategy. Notably, the most potent one, **14o** (IC_50_ = 0.0062 ± 0.0004 μM) was over 9-fold more potent than lead compound **13c** (IC_50_ = 0.059 ± 0.002 μM) and over 1419-fold more potent than positive control bestatin (IC_50_ = 8.8 ± 0.4 μM).

### 2.3. Anti-Proliferative Activities of Selected Compounds against Tumor Cells

Compound **14o,** the most potent against APN, was selected to further evaluate the anti-proliferative activities against six tumor cell lines (U937, K562, PLC/PRF/5, PC-3, ES-2, HepG2). As results listed in Table 2, compound **14o** exhibited much better anti-proliferative potencies than bestatin against all the tested tumor cell lines. Among them, K562 cells were most sensitive to compound **14o**.

### 2.4. Ex Vivo Anti-Angiogenesis Assay

**Figure 3:**
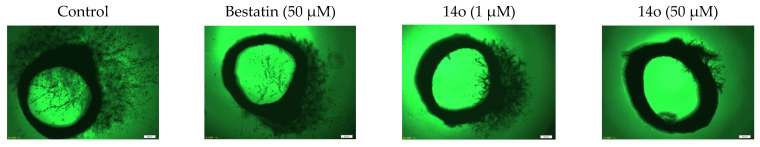
Representative images of the rat thoracic aorta rings treated with DMSO (0.5%) or compounds.

The anti-angiogenic potency of selected compound **14o** was preliminarily measured using the rat thoracic aorta ring model. As the results in Figure 3 show, compound **14o** could prevent the growth of micro-vessels derived from the thoracic aorta rings in a dose–dependent manner. Compared with the group treated with 1 μM of **14o**, obviously reduced micro-vessels were observed in the group treated with 50 μM of **14o**. Furthermore, at the same concentration of 50 μM, the anti-angiogenic activity of **14o** was much better than that of bestatin, which was consistent with the enzymatic inhibitory activities against APN. 

### 2.5. Docking Assay

**Figure 4:**
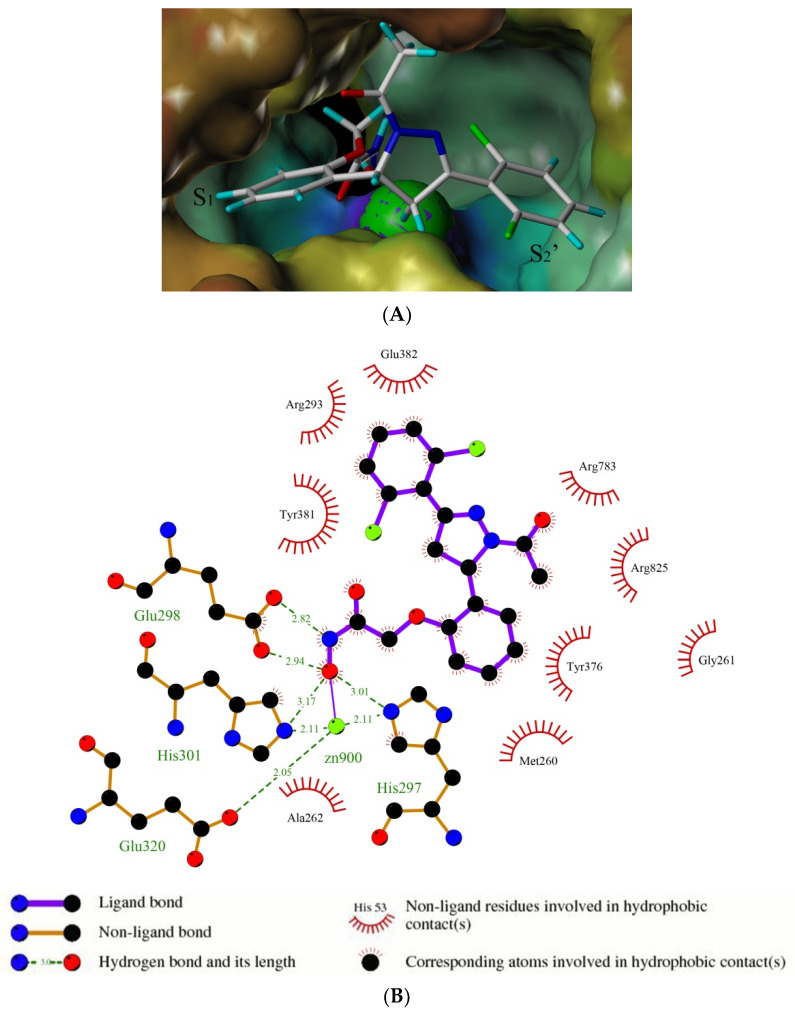
(**A**) The FlexX docking result of **14o** in the active site of APN (PDB code: 2DQM). The carbon atoms are marked in gray, oxygen atoms in red, hydrogen atoms in light blue, nitrogen atoms in blue and chlorine atoms in green. The green sphere represents zinc ion. S_1_ and S_2_′ represent two hydrophobic pockets. (**B**) The docking result shown by LIGPLOT. The carbon atoms, oxygen atoms, nitrogen atoms and chlorine atoms are marked in black, red, blue and green, respectively. The backbone of compound **14o** is labeled violet and the zinc ion is shown as green sphere. The hydrogen bonds are shown as dashed lines (distance in Å).

In order to investigate its binding mode in APN, compound **14o** was docked into the active site of APN (PDB code: 2DQM) using Sybyl 2.1. As depicted in Figure 4A, the terminal phenyl group with 2,6-diclorine substitution and another phenyl group occupied the S_2_′ and S_1_ hydrophobic pockets, respectively. Meanwhile, the hydroxamate moiety chelated the zinc ion. Moreover, one more detailed binding mode of compound **14o** with APN was illustrated in Figure 4B, generated with LIGPLOT. The terminal phenyl group could form hydrophobic interactions with the Arg^293^, Tyr^381^ and Glu^382^. Meanwhile, another phenyl group could form hydrophobic interactions with Met^260^ and Tyr^376^. In addition, the CH_3_CO group hydrophobically interacted with Arg^783^ and Arg^825^. Except for chelating the zinc ion, the hydroxamate moiety could form four hydrogen bonds with His^297^, Glu^298^ and His^301^ to enhance the potency of the interaction with APN.

## 3. Materials and Methods

### 3.1. Chemistry: General Procedures

All the commercially available materials were used without further purification otherwise noted. Thin-layer chromatography (TLC) was used for the monitoring of all the reactions on 0.25 mm silica gel plates (60 GF-254) and the product spots were visualized by ferric chloride, iodine vapor and UV light. The purification of the product was carried out by column chromatography. Melting points were determined on an electrothermal melting point apparatus without correction. ^1^H-NMR spectra were conducted on a Brucker DRX spectrometer with TMS as an internal standard. The values of chemical shifts were described as *δ* in parts per million and *J* in Hertz. HRMS were conducted by the Shandong Analysis and Test Center.

#### 3.1.1. General Procedure for Synthesis of Compounds **17t**–**17w** 1-(2-(Benzyloxy)phenyl)ethan-1-one (**17t**)

To a solution of 2-hydroxyacetophenone **16** (10.88 g, 80.00 mmol) in DMF was added the 60% NaH (3.84 g, 96.00 mmol), gradually. After stirring at 0 °C for 5 min, benzyl bromide (20.52 g, 120.00 mmol) was added into the mixture. The mixture was stirred at 25 °C for 12 h, then poured into water, followed by extraction with EtOAc. The organic phase was washed with brine and dried over MgSO_4_. After filtration and concentration under vacuum, the residue was purified by column chromatography (PE/EtOAc = 10:1, *v*/*v*) to give 16.63 g of compound **17t** as oil. Yield: 92%; ESI-MS m/z 227.1 [M+H]^+^.

Compounds **17u**–**17w** were prepared following the procedure described for compound **17t**.

#### 3.1.2. General Procedure for Synthesis of Compounds **18i**, **18l**, **18o**–**18w**

(E)-3-(5-Bromo-2-hydroxyphenyl)-1-(2-iodophenyl)prop-2-en-1-one (**18q**)

5-Bromo-2-hydroxybenzaldehyde **15d** (10.00 g, 50.00 mmol) and 2-iodoacetophen- one (14.76 g, 60.00 mmol) were dissolved in ethanol (200 mL), followed by the addition of potassium hydroxide aqueous solution (25.20 g, 450.00 mmol, in 50 mL water) at 0 °C. After stirring at 25 °C for 48 h, water was added into the mixture and pH was adjusted to 7 by 1N HCl. The mixture stood for 6 h. After filtration the residue was further purified by column chromatograph (DCM/MeOH = 100:5, *v/v*) to give 11.77 g of compound **18q** as a yellow solid. Yield: 55%, mp: 160–162 °C. ^1^H NMR (400 MHz, DMSO-*d_6_*): *δ* 10.65 (s, 1H), 7.97 (d, *J* = 7.9 Hz, 1H), 7.94–7.92 (m, 1H), 7.56–7.54 (m, 1H), 7.52–7.45 (m, 2H), 7.42 (dd, *J* = 8.8 Hz, *J* = 2.5 Hz, 1H), 7.32–7.21 (m, 2H), 6.87 (d, *J* = 8.8 Hz, 1H). ESI-MS *m*/*z* 428.5 [M+H] ^+^.

Compounds **18a**–**18h**, **18j**, **18k**, **18m** and **18n** were reported before [30]. Compounds **18i**, **18l**, **18o**, **18p** and **18r**–**18w** were prepared following the procedure described for the compound **18q**.

#### 3.1.3. General Procedure for Synthesis of Compounds **19a**–**19i**, **19k**–**19m**, **19o**–**19q**, **19s**, **19u**, **19w**, **19y**, **19aa**, **19cc**, **19ee** and **19gg**

1-(5-(2-Hydroxyphenyl)-3-(2-methoxyphenyl)-4,5-dihydro-1H-pyrazol-1-yl)ethan-1-one (**19d**) 

To a solution of **18d** (5.04 g, 20.00 mmol) in hot acetic acid (25 mL) was added 80% hydrazine hydrate (5.00 g, 80.00 mmol). After stirring at 120 °C for 5 h, the mixture was poured into water and the pH was adjusted to 7 by sodium bicarbonate. The formed precipitate was filtered off, dried at 50 °C and washed with EtOAc to give 4.03 g of compound **19d** as a white solid. Yield: 65%, mp: 206–208 °C. ^1^H NMR (400 MHz, DMSO-*d*_6_): *δ* 9.65 (s, 1H), 7.83 (dd, *J* = 7.8 Hz, *J* = 1.8 Hz, 1H), 7.43 (td, *J* = 7.8 Hz, *J* = 1.8 Hz, 1H), 7.11–6.99 (m, 3H), 6.81 (td, *J* = 7.8 Hz, *J* = 1.4 Hz, 2H), 6.73–6.69 (m, 1H), 5.59 (dd, *J* = 11.7 Hz, *J* = 4.2 Hz, 1H), 3.84 (dd, *J* = 18.4 Hz, *J* = 11.7 Hz, 1H), 3.78 (s, 3H), 3.02 (dd, *J* = 18.4 Hz, *J* = 4.2 Hz, 1H), 2.31 (s, 3H). ESI-MS *m*/*z* 311.2 [M+H] ^+^.

Compounds **19a**–**19c**, **19e**–**19i**, **19k**–**19m**, **19o**–**19q**, **19s**, **19u**, **19w**, **19y**, **19aa**, **19cc**, **19ee** and **19gg** were prepared following the procedure described for the compound **19d**.

#### 3.1.4. General Procedure for Synthesis of Compounds **19j**, **19n**, **19r**, **19t**, **19v**, **19x**, **19z**, **19bb**, **19dd**, **19ff** and **19hh**

5-(2-Hydroxyphenyl)-3-(2-iodophenyl)-4,5-dihydro-1H-pyrazole-1-carboxamide (**19j**)

Semicarbazide hydrochloride (2.68 g, 24.00 mmol) and sodium hydroxide (3.60 g, 90.00 mmol) were added to the solution of **18i** (7.00 g, 20.00 mmol) in ethanol (100 mL). After stirring at 78 °C for 5 h, the mixture was poured into water and the excess base was neutralized by 10% HCl. The formed precipitate was filtered off, dried at 50 °C and washed with EtOAc to give 5.54 g of compound **19j** as a white solid. Yield: 68%, mp: 176–178 °C. ESI-MS *m*/*z* 408.3 [M+H]^+^.

Compounds **19n**, **19r**, **19t**, **19v**, **19x**, **19z**, **19bb**, **19dd**, **19ff** and **19hh** were prepared following the procedure described for the compound **19j**.

#### 3.1.5. General Procedure for Synthesis of Compounds **20a**–**20z** and **20aa**–**20hh**

Methyl 2-(2-(1-acetyl-3-(2-methoxyphenyl)-4,5-dihydro-1H-pyrazol-5-yl)phenoxy) acetate (**20d**)

To a solution of compound **19d** (1.50 g, 4.84 mmol) in DMF (20 mL) was added 60% NaH (0.23 g, 5.81 mmol) gradually. After stirring at 25 °C for 5 min, methyl bromoacetate (1.11 g, 7.26 mmol) was added. Then the mixture was stirred at 25 °C for 12 h, followed by the addition of water (100 mL). The formed precipitate was filtered off and further purified by column chromatography (DCM/MeOH = 50:1, *v/v*) to give 1.04 g of compound **20d** as a white solid. Yield: 56%, mp: 126–128 °C. ^1^H NMR (400 MHz, DMSO-*d*_6_): *δ* 7.81 (dd, *J* = 7.8 Hz, *J* = 1.8 Hz, 1H), 7.43 (td, *J* = 7.8 Hz, *J* = 1.8 Hz, 1H), 7.22–7.18 (m, 1H), 7.10 (d, *J* = 8.3 Hz, 1H), 7.01 (t, *J* = 7.5 Hz, 1H), 6.95 (d, *J* = 7.8 Hz, 1H), 6.91–6.90 (m, 2H), 5.67 (dd, *J* = 11.7 Hz, *J* = 4.2 Hz, 1H), 4.95–4.86 (m, 2H), 3.85 (dd, *J* = 18.5 Hz, *J* = 11.7 Hz, 1H), 3.78 (s, 3H), 3.69 (s, 3H), 3.15 (dd, *J* = 18.5 Hz, *J* = 4.2 Hz, 1H), 2.32 (s, 3H). ESI-MS *m*/*z* 383.5 [M+H] ^+^.

Compounds **20a**–**20c**, **20e**–**20z** and **20aa**–**20hh** were prepared following the procedure described for the compound **20d**.

#### 3.1.6. General Procedure for Synthesis of Compounds **14a**–**14z** and **14aa**–**14hh**

2-(2-(1-Acetyl-3-(2-bromophenyl)-4,5-dihydro-1H-pyrazol-5-yl)phenoxy)-N-hydroxy acetamide (**14a**)

A solution of potassium hydroxide (15.00 g, 267.86 mmol) in anhydrous methanol (50 mL) was dropwise added into a solution of hydroxylamine hydrochloride (12.54 g, 180.43 mmol) in anhydrous methanol (50 mL) at 0 °C. After stirring at 0 °C for 40 min, the mixture was filtered and the filtrate was used for the transformation of the methyl ester group to a hydroxamate group. Compound **20a** (1.00 g, 2.32 mmol) was dissolved in the above solution (10 mL) and the mixture was stirred at 25 °C for 0.5 h. Then the mixture was poured into water (100 mL) and 10% H_3_PO_4_ solution was added to neutralize the excess base. The formed precipitate was filtered off and dried under vacuum to give 0.54 g of compound **14a** as a white solid. Yield: 54%, mp: 162–164 °C. ^1^H NMR (400 MHz, DMSO-*d*_6_): *δ* 10.76 (s, 1H), 9.04 (s, 1H), 7.81 (dd, *J* = 7.8 Hz, *J* = 1.8 Hz, 1H), 7.45–7.41 (m, 1H), 7.24–7.20 (m, 1H), 7.10 (d, *J* = 8.4 Hz, 1H), 7.03–6.96 (m, 2H), 6.94–6.86 (m, 2H), 5.81 (dd, *J* = 11.7 Hz, *J* = 4.3 Hz, 1H), 4.62–4.54 (m, 2H), 3.88 (dd, *J* = 18.6 Hz, *J* = 11.7 Hz, 1H), 3.79 (s, 3H), 3.14 (dd, *J* = 18.6 Hz, *J* = 4.3 Hz, 1H), 2.32 (s, 3H); ^13^C NMR (100 MHz, DMSO-*d*_6_): *δ* 168.25, 164.76, 155.12, 154.77, 134.51, 132.62, 131.73, 131.47, 129.88, 128.89, 128.37, 126.02, 121.65, 121.14, 112.69, 66.53, 55.37, 44.23, 22.24; HRMS (AP-ESI) *m*/*z* [M+H]^+^ calcd for C_19_H_19_BrN_3_O_4_: 432.0559, found: 432.0548.

Compounds **14b**–**14z** and **14aa**–**14hh** were prepared following the procedure described for the compound **14a**.

2-(2-(1-Acetyl-3-(3-bromophenyl)-4,5-dihydro-1H-pyrazol-5-yl)phenoxy)-N-hydroxy acetamide (**14b**)

White solid, yield: 55%, mp: 152–154 °C. ^1^H NMR (400 MHz, DMSO-*d*_6_): *δ* 10.74 (s, 1H), 9.04 (s, 1H), 7.91 (t, *J* = 1.8 Hz, 1H), 7.76 (d, *J* = 7.8 Hz, 1H), 7.66 (dd, *J* = 7.8 Hz, *J* = 2.0 Hz, 1H), 7.42 (t, *J* = 7.9 Hz, 1H), 7.26–7.20 (m, 1H), 6.98 (d, *J* = 8.3 Hz, 1H), 6.91 (d, *J* = 4.5 Hz, 2H), 5.85 (dd, *J* = 11.8 Hz, *J* = 4.6 Hz, 1H), 4.62 (d, *J* = 14.2 Hz, 1H), 4.55 (d, *J* = 14.2 Hz, 1H), 3.80 (dd, *J* = 18.2 Hz, *J* = 11.8 Hz, 1H), 3.21 (dd, *J* = 18.2 Hz, *J* = 4.6 Hz, 1H), 2.36 (s, 3H); ^13^C NMR (100 MHz, DMSO-*d*_6_): *δ* 168.07, 164.78, 154.73, 154.28, 134.01, 133.32, 131.40, 129.95, 129.34, 128.88, 126.05, 125.84, 122.57, 121.63, 112.72, 66.67, 55.62, 41.37, 22.21; HRMS (AP-ESI) *m*/*z* [M+H]^+^ calcd for C_19_H_19_BrN_3_O_4_: 432.0559, found: 432.0549.

2-(2-(1-Acetyl-3-(4-bromophenyl)-4,5-dihydro-1H-pyrazol-5-yl)phenoxy)-N-hydroxy acetamide (**14c**)

White solid, yield: 55%, mp: 202–204 °C. ^1^H NMR (400 MHz, DMSO-*d*_6_): *δ* 10.75 (s, 1H), 9.04 (s, 1H), 7.71–7.65 (m, 4H), 7.26–7.20 (m, 1H), 6.98 (d, *J* = 8.3 Hz, 1H), 6.91 (d, *J* = 4.5 Hz, 2H), 5.85 (dd, *J* = 11.8 Hz, *J* = 4.6 Hz, 1H), 4.62 (d, *J* = 14.2 Hz, 1H), 4.54 (d, *J* = 14.2 Hz, 1H), 3.81 (dd, *J* = 18.1 Hz, *J* = 11.8 Hz, 1H), 3.19 (dd, *J* = 18.1 Hz, *J* = 4.6 Hz, 1H), 2.35 (s, 3H); ^13^C NMR (100 MHz, DMSO-*d*_6_): *δ* 167.96, 164.75, 154.73, 154.66, 132.23, 130.89, 129.99, 128.97, 128.86, 125.87, 124.09, 121.63, 112.71, 66.61, 55.59, 41.41, 22.19; HRMS (AP-ESI) *m*/*z* [M+H]^+^ calcd for C_19_H_19_BrN_3_O_4_: 432.0559, found: 432.0563.

2-(2-(1-Acetyl-3-(2-methoxyphenyl)-4,5-dihydro-1H-pyrazol-5-yl)phenoxy)-N-hydroxy acetamide (**14d**)

White solid, yield: 52%, mp: 170–172 °C. ^1^H NMR (400 MHz, DMSO-*d*_6_): *δ* 10.76 (s, 1H), 9.04 (s, 1H), 7.81 (dd, *J* = 7.8 Hz, *J* = 1.8 Hz, 1H), 7.45–7.41 (m, 1H), 7.24–7.20 (m, 1H), 7.10 (d, *J* = 8.4 Hz, 1H), 7.03–6.96 (m, 2H), 6.94–6.86 (m, 2H), 5.81 (dd, *J* = 11.7 Hz, *J* = 4.3 Hz, 1H), 4.62–4.54 (m, 2H), 3.88 (dd, *J* = 18.6 Hz, *J* = 11.7 Hz, 1H), 3.79 (s, 3H), 3.14 (dd, *J* = 18.6 Hz, *J* = 4.3 Hz, 1H), 2.32 (s, 3H); ^13^C NMR (100 MHz, DMSO-*d*_6_): *δ* 167.82, 164.77, 158.45, 154.94, 154.64, 132.17, 130.42, 129.09, 128.72, 125.62, 121.68, 121.08, 120.57, 112.85, 112.62, 66.52, 56.18, 54.87, 44.94, 22.20; HRMS (AP-ESI) *m*/*z* [M+H]^+^ calcd for C_20_H_22_N_3_O_5_: 384.1559, found: 384.1552.

2-(2-(1-Acetyl-3-(3-methoxyphenyl)-4,5-dihydro-1H-pyrazol-5-yl)phenoxy)-N-hydroxy acetamide (**14e**)

White solid, yield: 57%, mp: 170–172 °C. ^1^H NMR (400 MHz, DMSO-*d*_6_): *δ* 10.76 (s, 1H), 9.06 (s, 1H), 7.40–7.32 (m, 2H), 7.28–7.20 (m, 2H), 7.06–6.98 (m, 2H), 6.94–6.89 (m, 2H), 5.85 (dd, *J* = 11.7 Hz, *J* = 4.4 Hz, 1H), 4.63 (d, *J* = 14.3 Hz, 1H), 4.56 (d, *J* = 14.3 Hz, 1H), 3.84–3.77 (m, 4H), 3.20 (dd, *J* = 18.1 Hz, *J* = 4.4 Hz, 1H), 2.36 (s, 3H); ^13^C NMR (100 MHz, DMSO-*d*_6_): *δ* 167.90, 164.78, 159.83, 155.47, 154.70, 132.98, 130.37, 130.12, 128.82, 125.74, 121.65, 119.51, 116.56, 112.69, 112.03, 66.65, 55.68, 55.30, 41.65, 22.17; HRMS (AP-ESI) *m*/*z* [M+H]^+^ calcd for C_20_H_22_N_3_O_5_: 384.1559, found: 384.1552.

2-(2-(1-Acetyl-3-(4-methoxyphenyl)-4,5-dihydro-1H-pyrazol-5-yl)phenoxy)-N-hydroxy acetamide (**14f**)

White solid, yield: 55%, mp: 120–122 °C. ^1^H NMR (400 MHz, DMSO-*d*_6_): *δ* 10.76 (s, 1H), 9.06 (s, 1H), 7.70 (d, *J* = 8.6 Hz, 2H), 7.25–7.19 (m, 1H), 7.02–6.97 (m, 3H), 6.93–6.85 (m, 2H), 5.83 (dd, *J* = 11.6 Hz, *J* = 4.2 Hz, 1H), 4.63 (d, *J* = 14.3 Hz, 1H), 4.56 (d, *J* =14.3 Hz, 1H), 3.79 (s, 3H), 3.78 (dd, *J* = 18.2 Hz, *J* = 11.6 Hz, 1H), 3.15 (dd, *J* = 18.2 Hz, *J* = 4.2 Hz, 1H), 2.34 (s, 3H); ^13^C NMR (100 MHz, DMSO-*d*_6_): *δ* 167.60, 164.79, 161.36, 155.35, 154.69, 130.23, 128.77, 128.71, 125.67, 124.12, 121.64, 114.65, 112.64, 66.62, 55.80, 55.04, 41.71, 22.17; HRMS (AP-ESI) *m*/*z* [M+H]^+^ calcd for C_20_H_22_N_3_O_5_: 384.1559, found: 384.1564.

2-(2-(1-Acetyl-3-(2-fluorophenyl)-4,5-dihydro-1H-pyrazol-5-yl)phenoxy)-N-hydroxy acetamide (**14g**)

White solid, yield: 50%, mp: 176–178 °C. ^1^H NMR (400 MHz, DMSO-*d*_6_): *δ* 10.75 (s, 1H), 9.03 (s, 1H), 7.90–7.85 (m, 1H), 7.54–7.48 (m, 1H), 7.33–7.29 (m, 2H), 7.27–7.20 (m, 1H), 6.99–6.91 (m, 3H), 5.84 (dd, *J* = 11.8 Hz, *J* = 4.5 Hz, 1H), 4.62–4.53 (m, 2H), 3.93–3.85 (m, 1H), 3.20–3.13 (m, 1H), 2.34 (s, 3H); ^13^C NMR (100 MHz, DMSO-*d*_6_): *δ* 168.12, 164.79, 159.46, 154.73, 151.82, 132.70, 130.02, 129.53, 128.88, 125.83, 125.28, 121.66, 119.69, 117.18, 112.67, 66.51, 55.08, 43.74, 22.15; HRMS (AP-ESI) *m*/*z* [M+H]^+^ calcd for C_19_H_19_FN_3_O_4_: 372.1360, found: 372.1365.

2-(2-(1-Acetyl-3-(2-chlorophenyl)-4,5-dihydro-1H-pyrazol-5-yl)phenoxy)-N-hydroxy acetamide (**14h**)

White solid, yield: 54%, mp: 166–168 °C. ^1^H NMR (400 MHz, DMSO-*d*_6_): *δ* 10.74 (s, 1H), 9.02 (s, 1H), 7.72 (dd, *J* = 7.5 Hz, *J* = 2.1 Hz, 1H), 7.55 (dd, *J* = 7.7 Hz, *J* = 1.6 Hz, 1H), 7.48–7.40 (m, 2H), 7.26–7.22 (m, 1H), 6.99–6.91 (m, 3H), 5.84 (dd, *J* = 11.8 Hz, *J* = 4.5 Hz, 1H), 4.62–4.53 (m, 2H), 3.95 (dd, *J* = 18.1 Hz, *J* = 11.8 Hz, 1H), 3.21 (dd, *J* = 18.1 Hz, *J* = 4.5 Hz, 1H), 2.32 (s, 3H); ^13^C NMR (100 MHz, DMSO-*d*_6_): *δ* 168.25, 164.80, 154.78, 154.25, 132.12, 131.64, 131.27, 131.17, 130.66, 129.90, 128.92, 127.90, 125.98, 121.68, 112.70, 66.53, 55.37, 44.25, 22.20; HRMS (AP-ESI) *m*/*z* [M+H]^+^ calcd for C_19_H_19_ClN_3_O_4_: 388.1064, found: 388.1072.

2-(2-(1-Acetyl-3-(2-iodophenyl)-4,5-dihydro-1H-pyrazol-5-yl)phenoxy)-N-hydroxy acetamide (**14i**)

White solid, yield: 58%, mp: 174–176 °C. ^1^H NMR (400 MHz, DMSO-*d*_6_): *δ* 10.92 (s, 1H), 9.02 (s, 1H), 8.04 (d, *J* = 7.8 Hz, 1H), 7.58–7.47 (m, 3H), 7.21–7.07 (m, 3H), 5.89 (dd, *J* = 12.0 Hz, *J* = 5.4 Hz, 1H), 4.64 (d, *J* = 13.2 Hz, 1H), 4.43 (d, *J* = 13.2 Hz, 1H), 3.94 (dd, *J* = 18.3 Hz, *J* = 12.0 Hz, 1H), 3.16 (dd, *J* = 18.3 Hz, *J* = 5.4 Hz, 1H), 2.31 (s, 3H); ^13^C NMR (100 MHz, DMSO-*d*_6_): *δ* 168.05, 164.83, 156.19, 154.82, 137.70, 132.03, 130.45, 130.15, 129.88, 129.79, 128.82, 126.53, 125.79, 121.67, 112.70, 66.60, 54.37, 43.76, 23.37, 22.24; HRMS (AP-ESI) *m*/*z* [M+H]^+^ calcd for C_19_H_19_IN_3_O_4_: 480.0420, found: 480.0418.

5-(2-(2-(Hydroxyamino)-2-oxoethoxy)phenyl)-3-(2-iodophenyl)-4,5-dihydro-1H-pyrazole-1-carboxamide (**14j**)

White solid, yield: 51%, mp: 164–166 °C. ^1^H NMR (400 MHz, DMSO-*d*_6_): *δ* 10.93 (s, 1H), 9.01 (s, 1H), 8.00 (d, *J* = 7.9 Hz, 1H), 7.57–7.52 (m, 2H), 7.50–7.46 (m, 1H), 7.23–7.11 (m, 3H), 6.48 (s, 2H), 5.77 (dd, *J* = 12.2 Hz, *J* = 6.2 Hz, 1H), 4.64 (d, *J* = 13.1 Hz, 1H), 4.41 (d, *J* = 13.1 Hz, 1H), 3.92 (dd, *J* = 18.1 Hz, *J* = 12.2 Hz, 1H), 3.12 (dd, *J* = 18.1 Hz, *J* = 6.2 Hz, 1H); ^13^C NMR (100 MHz, DMSO-*d*_6_): *δ* 164.84, 155.54, 154.59, 153.22, 141.08, 136.17, 131.32, 131.27, 130.80, 128.77, 128.72, 126.20, 121.66, 112.44, 95.70, 66.56, 55.10, 44.46; HRMS (AP-ESI) *m*/*z* [M+H]^+^ calcd for C_18_H_18_IN_4_O_4_: 481.0373, found: 481.0371.

2-(2-(1-Acetyl-3-(o-tolyl)-4,5-dihydro-1H-pyrazol-5-yl)phenoxy)-N-hydroxyacetamide (**14k**)

White solid, yield: 50%, mp: 170–172 °C. ^1^H NMR (400 MHz, DMSO-*d*_6_): *δ* 10.75 (s, 1H), 9.03 (s, 1H), 7.47 (d, *J* = 7.6 Hz, 1H), 7.33 (d, *J* = 4.3 Hz, 2H), 7.29–7.21 (m, 2H), 6.99–6.92 (m, 3H), 5.78 (dd, *J* = 11.7 Hz, *J* = 4.4 Hz, 1H), 4.64–4.54 (m, 2H), 3.88 (dd, *J* = 17.8 Hz, *J* = 11.7 Hz, 1H), 3.22 (dd, *J* = 17.8 Hz, *J* = 4.4 Hz, 1H), 2.61 (s, 3H), 2.33 (s, 3H); ^13^C NMR (100 MHz, DMSO-*d*_6_): *δ* 168.05, 164.83, 156.19, 154.82, 137.70, 132.03, 130.45, 130.15, 129.88, 129.79, 128.82, 126.53, 125.79, 121.67, 112.70, 66.60, 54.37, 43.76, 23.37, 22.24; HRMS (AP-ESI) *m*/*z* [M+H]^+^ calcd for C_20_H_22_N_3_O_4_: 368.1610, found: 368.1607.

2-(2-(1-Acetyl-3-(2,4-dichlorophenyl)-4,5-dihydro-1H-pyrazol-5-yl)phenoxy)-N-hydroxyacetamide (**14l**)

White solid, yield: 53%, mp: 128–130 °C. ^1^H NMR (400 MHz, DMSO-*d*_6_): *δ* 10.67 (s, 1H), 8.95 (s, 1H), 7.68–7.64 (m, 2H), 7.43 (dd, *J* = 8.6 Hz, *J* = 2.1 Hz, 1H), 7.17–7.13 (m, 1H), 6.90–6.82 (m, 3H), 5.74 (dd, *J* = 11.7 Hz, *J* = 4.6 Hz, 1H), 4.52 (d, *J* = 14.2 Hz, 1H), 4.45 (d, *J* = 14.2 Hz, 1H), 3.86 (dd, *J* = 18.1 Hz, *J* = 11.7 Hz, 1H), 3.14 (dd, *J* = 18.1 Hz, *J* = 4.6 Hz, 1H), 2.24 (s, 3H); ^13^C NMR (100 MHz, DMSO-*d*_6_): *δ* 168.28, 164.73, 154.78, 153.21, 135.30, 133.10, 132.40, 130.77, 129.76, 129.59, 128.93, 128.11, 126.10, 121.63, 112.70, 66.48, 55.57, 43.99, 22.20; HRMS (AP-ESI) *m*/*z* [M+H]^+^ calcd for C_19_H_18_Cl_2_N_3_O_4_: 422.0674, found: 422.0675.

2-(2-(1-Acetyl-3-(2,6-dimethoxyphenyl)-4,5-dihydro-1H-pyrazol-5-yl)phenoxy)-N-hydroxyacetamide (**14m**)

White solid, yield: 55%, mp: 178–180 °C. ^1^H NMR (400 MHz, DMSO-*d*_6_): *δ* 10.70 (s, 1H), 8.98 (s, 1H), 7.36 (t, *J* = 8.4 Hz, 1H), 7.25–7.21 (m, 1H), 7.07 (d, *J* = 7.6 Hz, 1H), 7.01–6.95 (m, 2H), 6.71 (d, *J* = 8.4 Hz, 2H), 5.84 (dd, *J* = 11.7 Hz, *J* = 4.3 Hz, 1H), 4.62–4.53 (m, 2H), 3.73 (s, 3H), 3.66 (dd, *J* = 18.1 Hz, *J* = 11.7 Hz, 1H), 3.33 (s, 2H), 2.75 (dd, *J* = 18.1 Hz, *J* = 4.3 Hz, 1H), 2.21 (s, 3H); ^13^C NMR (100 MHz, DMSO-*d*_6_): *δ* 167.69, 164.84, 158.58, 154.43, 153.17, 131.56, 130.84, 128.78, 125.93, 121.87, 112.46, 110.02, 104.78, 66.70, 56.45, 53.69, 45.71, 22.22; HRMS (AP-ESI) *m*/*z* [M+H]^+^ calcd for C_21_H_24_N_3_O_6_: 414.1665, found: 414.1661.

3-(2,6-Dimethoxyphenyl)-5-(2-(2-(hydroxyamino)-2-oxoethoxy)phenyl)-4,5-dihydro-1H-pyrazole-1-carboxamide (**14n**)

White solid, yield: 52%, mp: 212–214 °C. ^1^H NMR (400 MHz, DMSO-*d*_6_): *δ* 10.74 (s, 1H), 8.94 (s, 1H), 7.35 (t, *J* = 8.4 Hz, 1H), 7.24–7.17 (m, 2H), 7.00 (t, *J* = 7.5 Hz, 1H), 6.94 (d, *J* = 8.3 Hz, 1H), 6.70 (d, *J* = 8.5 Hz, 2H), 6.33 (s, 2H), 5.73 (dd, *J* = 11.9 Hz, *J* = 4.8 Hz, 1H), 4.61 (d, *J* = 14.4 Hz, 1H), 4.54 (d, *J* = 14.4 Hz, 1H), 3.73 (s, 6H), 3.63 (dd, *J* = 17.9 Hz, *J* = 11.9 Hz, 1H), 2.72 (dd, *J* = 17.9 Hz, *J* = 4.8 Hz, 1H); ^13^C NMR (100 MHz, DMSO-*d*_6_): *δ* 164.88, 158.65, 155.48, 154.25, 149.53, 132.23, 131.35, 128.59, 126.36, 121.80, 112.09, 110.34, 104.65, 66.56, 56.37, 53.38, 45.88; HRMS (AP-ESI) *m*/*z* [M+H]^+^ calcd for C_20_H_23_N_4_O_6_: 415.1618, found: 415.1622.

2-(2-(1-Acetyl-3-(2,6-dichlorophenyl)-4,5-dihydro-1H-pyrazol-5-yl)phenoxy)-N-hydroxyacetamide (**14o**)

White solid, yield: 54%, mp: 174–176 °C. ^1^H NMR (600 MHz, DMSO-*d*_6_): *δ* 10.73 (s, 1H), 9.02 (s, 1H), 7.61–7.57 (m, 1H), 7.52 (t, *J* = 8.1 Hz, 1H), 7.29–7.20 (m, 1H), 7.04 (d, *J* = 7.7 Hz, 1H), 7.02–6.95 (m, 2H), 5.98 (dd, *J* = 11.9 Hz, *J* = 4.7 Hz, 1H), 4.60 (d, *J* = 14.2 Hz, 1H), 4.55 (d, *J* = 14.2 Hz, 1H), 3.82 (dd, *J* = 18.6 Hz, *J* = 11.9 Hz, 1H), 2.91 (dd, *J* = 18.6 Hz, *J* = 4.7 Hz, 1H), 2.28 (s, 3H); ^13^C NMR (150 MHz, DMSO-*d*_6_): *δ* 168.21, 164.73, 154.49, 153.23, 134.25, 132.58, 130.55, 129.99, 129.04, 128.91, 125.53, 121.70, 112.68, 66.72, 54.86, 44.88, 22.17; HRMS (AP-ESI) *m*/*z* [M+H]^+^ calcd for C_19_H_18_Cl_2_N_3_O_4_: 422.0674, found: 422.0668.

2-(2-(1-Acetyl-3-(naphthalen-1-yl)-4,5-dihydro-1H-pyrazol-5-yl)phenoxy)-N-hydroxy acetamide (**14p**)

White solid, yield: 55%, mp: 198–200 °C. ^1^H NMR (400 MHz, DMSO-*d*_6_): *δ* 10.81 (s, 1H), 9.25 (d, *J* = 8.7 Hz, 1H), 9.06 (s, 1H), 8.04–8.01 (m, 2H), 7.75 (d, *J* = 7.2 Hz, 1H), 7.72–7.68 (m, 1H), 7.63–7.60 (m, 1H), 7.55 (t, *J* = 7.7 Hz, 1H), 7.25 (td, *J* = 7.7 Hz, *J* = 1.8 Hz, 1H), 7.03–6.99 (m, 2H), 6.94 (t, *J* = 7.4 Hz, 1H), 5.86 (dd, *J* = 11.7 Hz, *J* = 4.3 Hz, 1H), 4.65 (d, *J* = 14.3 Hz, 1H), 4.58 (d, *J* = 14.3 Hz, 1H), 4.07 (dd, *J* = 17.8 Hz, *J* = 11.7 Hz, 1H), 3.42–3.38 (m, 1H), 2.45 (s, 3H); ^13^C NMR (100 MHz, DMSO-*d*_6_): *δ* 168.05, 164.82, 155.98, 154.88, 134.09, 131.38, 130.32, 130.09, 129.41, 129.27, 128.84, 128.17, 127.90, 126.77, 125.89, 125.66, 121.66, 112.70, 66.58, 54.27, 44.11, 22.43; HRMS (AP-ESI) *m*/*z* [M+H]^+^ calcd for C_23_H_22_N_3_O_4_: 404.1610, found: 404.1605.

2-(2-(1-Acetyl-3-(2-iodophenyl)-4,5-dihydro-1H-pyrazol-5-yl)-6-bromophenoxy)-N-hydroxyacetamide (**14q**)

White solid, yield: 50%, mp: 96–98 °C. ^1^H NMR (400 MHz, DMSO-*d*_6_): *δ* 10.92 (s, 1H), 9.02 (s, 1H), 8.04 (d, *J* = 7.8 Hz, 1H), 7.58–7.47 (m, 3H), 7.21–7.07 (m, 3H), 5.89 (dd, *J* = 12.0 Hz, *J* = 5.4 Hz, 1H), 4.64 (d, *J* = 13.2 Hz, 1H), 4.43 (d, *J* = 13.2 Hz, 1H), 3.94 (dd, *J* = 18.3 Hz, *J* = 12.0 Hz, 1H), 3.16 (dd, *J* = 18.3 Hz, *J* = 5.4 Hz, 1H), 2.31 (s, 3H); ^13^C NMR (100 MHz, DMSO-*d*_6_): *δ* 168.41, 164.17, 155.77, 151.90, 141.24, 138.41, 135.59, 132.92, 131.68, 130.96, 128.86, 127.46, 126.00, 116.95, 95.70, 70.77, 54.74, 44.92, 22.40; HRMS (AP-ESI) *m/z* [M+H]^+^ calcd for C_19_H_18_BrIN_3_O_4_: 557.9525, found: 557.9528.

5-(3-Bromo-2-(2-(hydroxyamino)-2-oxoethoxy)phenyl)-3-(2-iodophenyl)-4,5-dihydro-1H-pyrazole-1-carboxamide (**14r**)

White solid, yield: 52%, mp: 140–142 °C. ^1^H NMR (400 MHz, DMSO-*d*_6_): *δ* 10.93 (s, 1H), 9.01 (s, 1H), 8.00 (d, *J* = 7.9 Hz, 1H), 7.57–7.52 (m, 2H), 7.50–7.46 (m, 1H), 7.23–7.11 (m, 3H), 6.48 (s, 2H), 5.77 (dd, *J* = 12.2 Hz, *J* = 6.2 Hz, 1H), 4.64 (d, *J* = 13.1 Hz, 1H), 4.41 (d, *J* = 13.1 Hz, 1H), 3.92 (dd, *J* = 18.1 Hz, *J* = 12.2 Hz, 1H), 3.12 (dd, *J* = 18.1 Hz, *J* = 6.2 Hz, 1H); ^13^C NMR (100 MHz, DMSO-*d*_6_): *δ* 164.28, 155.45, 152.79, 151.72, 140.93, 139.58, 136.11, 132.66, 131.39, 130.94, 128.79, 127.34, 126.41, 116.93, 95.96, 70.81, 55.10, 45.27; HRMS (AP-ESI) *m*/*z* [M+H]^+^ calcd for C_18_H_17_BrIN_4_O_4_: 558.9478, found: 558.9482.

2-(2-(1-Acetyl-3-(2-iodophenyl)-4,5-dihydro-1H-pyrazol-5-yl)-5-bromophenoxy)-N-hydroxyacetamide (**14s**)

White solid, yield: 54%, mp: 168–170 °C. ^1^H NMR (400 MHz, DMSO-*d*_6_): *δ* 10.74 (s, 1H), 9.08 (s, 1H), 8.02 (d, *J* = 7.9 Hz, 1H), 7.54–7.43 (m, 2H), 7.19–7.10 (m, 3H), 6.96 (d, *J* = 8.2 Hz, 1H), 5.76 (dd, *J* = 11.8 Hz, *J* = 4.8 Hz, 1H), 4.68–4.58 (m, 2H), 3.88 (dd, *J* = 18.1 Hz, *J* = 11.8 Hz, 1H), 3.20 (dd, *J* = 18.1 Hz, *J* = 4.8 Hz, 1H), 2.34 (s, 3H); ^13^C NMR (100 MHz, DMSO-*d*_6_): *δ* 168.42, 164.43, 156.35, 155.72, 141.31, 135.66, 131.60, 130.81, 129.41, 128.83, 128.08, 124.45, 121.20, 116.03, 95.54, 66.71, 55.28, 43.69, 22.40; HRMS (AP-ESI) *m*/*z* [M+H]^+^ calcd for C_19_H_18_BrIN_3_O_4_: 557.9525, found: 557.9520.

5-(4-Bromo-2-(2-(hydroxyamino)-2-oxoethoxy)phenyl)-3-(2-iodophenyl)-4,5-dihydro-1H-pyrazole-1-carboxamide (**14t**)

White solid, yield: 57%, mp: 196–198 °C. ^1^H NMR (400 MHz, DMSO-*d*_6_): *δ* 10.76 (s, 1H), 9.04 (s, 1H), 7.99 (d, *J* = 7.9 Hz, 1H), 7.50–7.44 (m, 2H), 7.18–7.13 (m, 3H), 7.01 (d, *J* = 8.5 Hz, 1H), 6.49 (s, 2H), 5.68 (dd, *J* = 12.0 Hz, *J* = 5.4 Hz, 1H), 4.69–4.58 (m, 2H), 3.87 (dd, *J* = 18.0 Hz, *J* = 12.0 Hz, 1H), 3.13 (dd, *J* = 18.0 Hz, *J* = 5.4 Hz, 1H); ^13^C NMR (100 MHz, DMSO-*d*_6_): *δ* 164.48, 155.51, 155.48, 153.43, 141.00, 136.14, 131.32, 130.82, 130.79, 128.77, 128.04, 124.47, 120.96, 115.78, 95.80, 66.73, 55.06, 44.12; HRMS (AP-ESI) *m*/*z* [M+H]^+^ calcd for C_18_H_17_BrIN_4_O_4_: 558.9478, found: 558.9482.

2-(2-(1-Acetyl-3-(2-iodophenyl)-4,5-dihydro-1H-pyrazol-5-yl)-4-bromophenoxy)-N-hydroxyacetamide (**14u**)

White solid, yield: 51%, mp: 106–108 °C. ^1^H NMR (400 MHz, DMSO-*d*_6_): *δ* 10.76 (s, 1H), 9.04 (s, 1H), 8.03 (d, *J* = 7.9 Hz, 1H), 7.53–7.42 (m, 3H), 7.18 (t, *J* = 7.5 Hz, 1H), 7.11 (d, *J* = 2.5 Hz, 1H), 6.97 (d, *J* = 8.8 Hz, 1H), 5.79 (dd, *J* = 11.9 Hz, *J* = 4.9 Hz, 1H), 4.64–4.53 (m, 2H), 3.89 (dd, *J* = 18.2 Hz, *J* = 11.9 Hz, 1H), 3.23 (dd, *J* = 18.2 Hz, *J* = 4.9 Hz, 1H), 2.35 (s, 3H); ^13^C NMR (100 MHz, DMSO-*d*_6_): *δ* 168.58, 164.46, 156.34, 154.25, 141.36, 135.52, 132.41, 131.65, 131.49, 130.85, 128.87, 115.21, 113.23, 95.47, 66.72, 55.30, 43.74, 22.41; HRMS (AP-ESI) *m*/*z* [M+H]^+^ calcd for C_19_H_18_BrIN_3_O_4_: 557.9525, found: 557.9522.

5-(5-Bromo-2-(2-(hydroxyamino)-2-oxoethoxy)phenyl)-3-(2-iodophenyl)-4,5-dihydro-1H-pyrazole-1-carboxamide (**14v**)

White solid, yield: 54%, mp: 202–204 °C. ^1^H NMR (400 MHz, DMSO-*d*_6_): *δ* 10.76 (s, 1H), 9.02 (s, 1H), 8.00 (d, *J* = 7.9 Hz, 1H), 7.51–7.41 (m, 3H), 7.18–7.14 (m, 2H), 6.96 (d, *J* = 8.8 Hz, 1H), 6.53 (s, 2H), 5.71 (dd, *J* = 12.0 Hz, *J* = 5.4 Hz, 1H), 4.64–4.54 (m, 2H), 3.89 (dd, *J* = 18.0 Hz, *J* = 12.0 Hz, 1H), 3.15 (dd, *J* = 18.0 Hz, *J* = 5.4 Hz, 1H); ^13^C NMR (100 MHz, DMSO-*d*_6_): *δ* 164.48, 155.53, 154.04, 153.53, 141.04, 136.04, 133.76, 131.38, 131.23, 130.82, 128.81, 128.76, 115.01, 113.24, 95.72, 66.77, 55.16, 44.18; HRMS (AP-ESI) *m*/*z* [M+H]^+^ calcd for C_18_H_17_BrIN_4_O_4_: 558.9478, found: 558.9472.

2-(2-(1-Acetyl-3-(2-iodophenyl)-4,5-dihydro-1H-pyrazol-5-yl)-3-bromophenoxy)-N-hydroxyacetamide (**14w**)

White solid, yield: 55%, mp: 152–154 °C. ^1^H NMR (400 MHz, DMSO-*d*_6_): *δ* 10.65 (s, 1H), 8.98 (s, 1H), 8.08–8.02 (m, 1H), 7.61–7.59 (m, 1H), 7.53–7.45 (m, 1H), 7.24–7.06 (m, 3H), 6.93–6.82 (m, 1H), 6.28–5.86 (m, 1H), 4.66–4.45 (m, 2H), 3.93–3.73 (m, 1H), 3.61–3.23 (m, 1H), 2.26–2.20 (m, 3H); HRMS (AP-ESI) *m*/*z* [M+H]^+^ calcd for C_19_H_18_BrIN_3_O_4_: 557.9525, found: 557.9520.

5-(2-Bromo-6-(2-(hydroxyamino)-2-oxoethoxy)phenyl)-3-(2-iodophenyl)-4,5-dihydro-1H-pyrazole-1-carboxamide (**14x**)

White solid, yield: 59%, mp: 132–134 °C. ^1^H NMR (400 MHz, DMSO-*d*_6_): *δ* 10.62 (m, 1H), 8.99 (s, 1H), 8.04–8.00 (m, 1H), 7.61–7.57 (m, 1H), 7.51–7.44 (m, 1H), 7.28–6.93 (m, 4H), 6.21–5.79 (m, 3H), 4.72–4.43 (m, 2H), 3.88–3.70 (m, 1H), 3.64–3.28 (m, 1H); HRMS (AP-ESI) *m/z* [M+H]^+^ calcd for C_18_H_17_BrIN_4_O_4_: 558.9478, found: 558.9484. 

2-(2-(1-Acetyl-3-(2-iodophenyl)-4,5-dihydro-1H-pyrazol-5-yl)-3-chlorophenoxy)-N-hydroxyacetamide (**14y**)

White solid, yield: 56%, mp: 168–170 °C. ^1^H NMR (400 MHz, DMSO-*d*_6_): *δ* 10.68–10.63 (m, 1H), 9.01 (s, 1H), 8.08–8.03 (m, 1H), 7.61–7.46 (m, 2H), 7.30–7.15 (m, 2H), 7.08–6.88 (m, 2H), 6.26–5.89 (m, 1H), 4.68–4.45 (m, 2H), 3.95–3.73 (m, 1H), 3.64–3.24 (m, 1H), 2.25–2.21 (m, 3H); HRMS (AP-ESI) *m*/*z* [M+H]^+^ calcd for C_19_H_18_ClIN_3_O_4_: 514.0031, found: 514.0035.

5-(2-Chloro-6-(2-(hydroxyamino)-2-oxoethoxy)phenyl)-3-(2-iodophenyl)-4,5-dihydro-1H-pyrazole-1-carboxamide (**14z**)

White solid, yield: 57%, mp: 190–192 °C. ^1^H NMR (400 MHz, DMSO-*d*_6_): *δ* 10.62 (s, 1H), 8.97 (s, 1H), 8.04–8.00 (m, 1H), 7.59–7.56 (m, 1H), 7.51–7.44 (m, 1H), 7.28–7.00 (m, 4H), 6.19–5.83 (m, 3H), 4.73–4.44 (m, 2H), 3.90–3.70 (m, 1H), 3.67–3.28 (m, 1H); HRMS (AP-ESI) *m*/*z* [M+H]^+^ calcd for C_18_H_17_ClIN_4_O_4_: 514.9983, found: 514.9987.

2-(2-(1-Acetyl-3-(2-(benzyloxy)phenyl)-4,5-dihydro-1H-pyrazol-5-yl)phenoxy)-N-hydroxyacetamide (**14aa**)

White solid, yield: 55%, mp: 162–164 °C. ^1^H NMR (400 MHz, DMSO-*d*_6_): *δ* 10.74 (s, 1H), 9.02 (s, 1H), 7.77 (d, *J* = 7.8 Hz, 1H), 7.44–7.39 (m, 3H), 7.36–7.28 (m, 3H), 7.24–7.20 (m, 2H), 7.02 (t, *J* = 7.5 Hz, 1H), 6.95 (d, *J* = 8.3 Hz, 1H), 6.92–6.86 (m, 2H), 5.79 (dd, *J* = 11.6 Hz, *J* = 4.2 Hz, 1H), 5.15 (s, 2H), 4.57 (d, *J* = 14.2 Hz, 1H), 4.47 (d, *J* = 14.2 Hz, 1H), 3.82 (dd, *J* = 18.5 Hz, *J* = 11.6 Hz, 1H), 3.18 (dd, *J* = 18.5 Hz, *J* = 4.2 Hz, 1H), 2.29 (s, 3H); ^13^C NMR (100 MHz, DMSO-*d*_6_): *δ* 167.88, 164.76, 157.33, 154.92, 154.61, 136.97, 132.05, 130.28, 129.54, 128.90, 128.76, 128.34, 128.14, 125.58, 121.66, 121.32, 121.01, 114.02, 112.57, 70.37, 66.53, 54.82, 44.91, 22.19; HRMS (AP-ESI) *m*/*z* [M+H]^+^ calcd for C_26_H_26_N_3_O_5_: 460.1872, found: 460.1868.

3-(2-(Benzyloxy)phenyl)-5-(2-(2-(hydroxyamino)-2-oxoethoxy)phenyl)-4,5-dihydro-1H-pyrazole-1-carboxamide (**14bb**)

White solid, yield: 52%, mp: 176–178 °C. ^1^H NMR (400 MHz, DMSO-*d*_6_): *δ* 10.75 (s, 1H), 8.99 (s, 1H), 7.88 (d, *J* = 7.7 Hz, 1H), 7.40–7.28 (m, 6H), 7.22–7.17 (m, 2H), 7.02–6.88 (m, 4H), 6.47 (s, 2H), 5.70 (dd, *J* = 11.9 Hz, *J* = 4.8 Hz, 1H), 5.17–5.10 (m, 2H), 4.58 (d, *J* = 14.4 Hz, 1H), 4.47 (d, *J* = 14.4 Hz, 1H), 3.80 (dd, *J* = 18.2 Hz, *J* = 11.9 Hz, 1H), 3.15 (dd, *J* = 18.2 Hz, *J* = 4.8 Hz, 1H); ^13^C NMR (100 MHz, DMSO-*d*_6_): *δ* 164.79, 157.09, 155.48, 154.40, 151.32, 137.04, 131.75, 131.55, 129.58, 128.92, 128.55, 128.35, 128.15, 125.74, 121.64, 121.34, 121.23, 113.93, 112.29, 70.34, 66.48, 54.54, 45.22; HRMS (AP-ESI) *m*/*z* [M+H]^+^ calcd for C_25_H_25_N_4_O_5_: 461.1825, found: 461.1830.

2-(2-(1-Acetyl-3-(2-((2-bromobenzyl)oxy)phenyl)-4,5-dihydro-1H-pyrazol-5-yl)phenoxy)-N-hydroxyacetamide (**14cc**)

White solid, yield: 53%, mp: 82–84 °C. ^1^H NMR (400 MHz, DMSO-*d*_6_): *δ* 10.68 (s, 1H), 9.04 (s, 1H), 7.79 (d, *J* = 7.7 Hz, 1H), 7.64 (d, *J* = 8.0 Hz, 1H), 7.58 (d, *J* = 7.6 Hz, 1H), 7.47–7.36 (m, 2H), 7.30 (t, *J* = 7.6 Hz, 1H), 7.23–7.19 (m, 2H), 7.06 (t, *J* = 7.5 Hz, 1H), 6.94 (d, *J* = 8.3 Hz, 1H), 6.90–6.84 (m, 2H), 5.79 (dd, *J* = 11.7 Hz, *J* = 4.2 Hz, 1H), 5.20–5.12 (m, 2H), 4.56 (d, *J* = 14.3 Hz, 1H), 4.45 (d, *J* = 14.3 Hz, 1H), 3.78 (dd, *J* = 18.5 Hz, *J* = 11.7 Hz, 1H), 3.16 (dd, *J* = 18.5 Hz, *J* = 4.2 Hz, 1H), 2.29 (s, 3H); ^13^C NMR (100 MHz, DMSO-*d*_6_): *δ* 167.82, 164.65, 157.14, 154.73, 154.54, 135.84, 133.12, 132.10, 130.92, 130.70, 130.18, 129.63, 128.70, 128.34, 125.51, 123.33, 121.59, 121.00, 113.91, 112.49, 70.31, 66.51, 54.73, 44.75, 22.15; HRMS (AP-ESI) *m*/*z* [M+H]^+^ calcd for C_26_H_25_BrN_3_O_5_: 538.0978, found: 538.0982.

3-(2-((2-Bromobenzyl)oxy)phenyl)-5-(2-(2-(hydroxyamino)-2-oxoethoxy)phenyl)-4,5-dihydro-1H-pyrazole-1-carboxamide (**14dd**)

White solid, yield: 56%, mp: 92–94 °C. ^1^H NMR (400 MHz, DMSO-*d*_6_): *δ* 10.67 (s, 1H), 9.03 (s, 1H), 7.90 (d, *J* = 7.8 Hz, 1H), 7.64 (d, *J* = 7.8 Hz, 1H), 7.55 (d, *J* = 7.5 Hz, 1H), 7.43–7.27 (m, 3H), 7.22–7.17 (m, 2H), 7.04 (t, *J* = 7.5 Hz, 1H), 6.94–6.87 (m, 3H), 6.49 (s, 2H), 5.70 (dd, *J* = 11.8 Hz, *J* = 4.8 Hz, 1H), 5.19–5.11 (m, 2H), 4.57 (d, *J* = 14.4 Hz, 1H), 4.45 (d, *J* = 14.4 Hz, 1H), 3.75 (dd, *J* = 18.4 Hz, *J* = 11.8 Hz, 1H), 3.13 (dd, *J* = 18.4 Hz, *J* = 4.8 Hz, 1H); ^13^C NMR (100 MHz, DMSO-*d*_6_): *δ* 164.68, 156.92, 155.41, 154.33, 151.15, 135.87, 133.14, 131.64, 131.60, 130.94, 130.72, 129.63, 128.51, 128.36, 125.69, 123.38, 121.57, 121.48, 121.34, 113.79, 112.19, 70.28, 66.44, 54.46, 45.04; HRMS (AP-ESI) *m*/*z* [M+H]^+^ calcd for C_25_H_24_BrN_4_O_5_: 539.0930, found: 539.0934.

2-(2-(1-Acetyl-3-(2-((3-bromobenzyl)oxy)phenyl)-4,5-dihydro-1H-pyrazol-5-yl)phenoxy)-N-hydroxyacetamide (**14ee**)

White solid, yield: 57%, mp: 164–166 °C. ^1^H NMR (400 MHz, DMSO-*d*_6_): *δ* 10.73 (s, 1H), 9.01 (s, 1H), 7.74 (dd, *J* = 7.8 Hz, *J* = 1.8 Hz, 1H), 7.66 (d, *J* = 2.2 Hz, 1H), 7.51 (d, *J* = 8.6 Hz, 1H), 7.43 (t, *J* = 7.4 Hz, 2H), 7.31 (t, *J* = 7.8 Hz, 1H), 7.23–7.18 (m, 2H), 7.03 (t, *J* = 7.5 Hz, 1H), 6.95 (d, *J* = 8.0 Hz, 1H), 6.89 (d, *J* = 7.4 Hz, 1H), 5.79 (dd, *J* = 11.6 Hz, *J* = 4.2 Hz, 1H), 5.17 (s, 2H), 4.58 (d, *J* = 14.4 Hz, 1H), 4.50 (d, *J* = 14.4 Hz, 1H), 3.85 (dd, *J* = 18.4 Hz, *J* = 11.6 Hz, 1H), 3.17 (dd, *J* = 18.4 Hz, *J* = 4.2 Hz, 1H), 2.29 (s, 3H); ^13^C NMR (100 MHz, DMSO-*d*_6_): *δ* 167.87, 164.78, 157.01, 154.69, 154.63, 139.89, 132.01, 131.18, 131.14, 130.75, 130.24, 129.69, 128.77, 127.07, 125.61, 122.11, 121.65, 121.49, 121.06, 114.03, 112.57, 69.40, 66.51, 54.74, 44.81, 22.20; HRMS (AP-ESI) *m*/*z* [M+H]^+^ calcd for C_26_H_25_BrN_3_O_5_: 538.0978, found: 538.0986.

3-(2-((3-Bromobenzyl)oxy)phenyl)-5-(2-(2-(hydroxyamino)-2-oxoethoxy)phenyl)-4,5-dihydro-1H-pyrazole-1-carboxamide (**14ff**)

White solid, yield: 59%, mp: 120–122 °C. ^1^H NMR (400 MHz, DMSO-*d*_6_): *δ* 10.74 (s, 1H), 8.98 (s, 1H), 7.87 (dd, *J* = 7.8 Hz, *J* = 1.8 Hz, 1H), 7.62 (t, *J* = 1.8 Hz, 1H), 7.51–7.49 (m, 1H), 7.41–7.36 (m, 2H), 7.30 (t, *J* = 7.8 Hz, 1H), 7.22–7.14 (m, 2H), 7.03–6.88 (m, 4H), 6.46 (s, 2H), 5.70 (dd, *J* = 11.9 Hz, *J* = 4.8 Hz, 1H), 5.16 (s, 2H), 4.59 (d, *J* = 14.4 Hz, 1H), 4.50 (d, *J* = 14.4 Hz, 1H), 3.81 (dd, *J* = 18.3 Hz, *J* = 11.9 Hz, 1H), 3.14 (dd, *J* = 18.3 Hz, *J* = 4.8 Hz, 1H); ^13^C NMR (100 MHz, DMSO-*d*_6_): *δ* 164.79, 156.81, 155.46, 154.40, 151.18, 139.92, 131.72, 131.54, 131.18, 130.80, 129.64, 128.56, 127.10, 125.77, 122.10, 121.64, 121.41, 113.97, 112.28, 69.36, 66.45, 54.53, 45.19; HRMS (AP-ESI) *m*/*z* [M+H]^+^ calcd for C_25_H_24_BrN_4_O_5_: 539.0930, found: 539.0936.

2-(2-(1-Acetyl-3-(2-((4-bromobenzyl)oxy)phenyl)-4,5-dihydro-1H-pyrazol-5-yl)phenoxy)-N-hydroxyacetamide (**14gg**)

White solid, yield: 53%, mp: 96–98 °C. ^1^H NMR (400 MHz, DMSO-*d*_6_): *δ* 10.75 (s, 1H), 9.02 (s, 1H), 7.74 (dd, *J* = 7.7 Hz, *J* = 1.8 Hz, 1H), 7.55–7.52 (m, 2H), 7.44–7.36 (m, 3H), 7.24–7.17 (m, 2H), 7.03 (t, *J* = 7.5 Hz, 1H), 6.95 (d, *J* = 8.3 Hz, 1H), 6.91–6.85 (m, 2H), 5.78 (dd, *J* = 11.6 Hz, *J* = 4.2 Hz, 1H), 5.13 (d, *J* = 1.8 Hz, 2H), 4.58 (d, *J* = 14.2 Hz, 1H), 4.50 (d, *J* = 14.2 Hz, 1H), 3.82 (dd, *J* = 18.4 Hz, *J* = 11.6 Hz, 1H), 3.18 (dd, *J* = 18.4 Hz, *J* = 4.2 Hz, 1H), 2.29 (s, 3H); ^13^C NMR (100 MHz, DMSO-*d*_6_): *δ* 167.86, 164.78, 157.11, 154.95, 154.64, 136.45, 132.03, 131.84, 130.37, 130.23, 129.62, 128.74, 125.63, 121.64, 121.54, 121.44, 121.10, 114.01, 112.54, 69.57, 66.48, 54.85, 44.81, 22.19; HRMS (AP-ESI) *m*/*z* [M+H]^+^ calcd for C_26_H_25_BrN_3_O_5_: 538.0978, found: 538.0980.

3-(2-((4-Bromobenzyl)oxy)phenyl)-5-(2-(2-(hydroxyamino)-2-oxoethoxy)phenyl)-4,5-dihydro-1H-pyrazole-1-carboxamide (**14hh**)

White solid, yield: 55%, mp: 96–98 °C. ^1^H NMR (400 MHz, DMSO-*d*_6_): *δ* 10.76 (s, 1H), 8.99 (s, 1H), 7.85 (dd, *J* = 7.8 Hz, *J* = 1.8 Hz, 1H), 7.53 (d, *J* = 8.3 Hz, 2H), 7.40–7.35 (m, 3H), 7.23–7.14 (m, 2H), 7.02–6.88 (m, 4H), 6.47 (s, 2H), 5.69 (dd, *J* = 11.9 Hz, *J* = 4.7 Hz, 1H), 5.16–5.09 (m, 2H), 4.59 (d, *J* = 14.4 Hz, 1H), 4.50 (d, *J* = 14.4 Hz, 1H), 3.80 (dd, *J* = 18.3 Hz, *J* = 11.9 Hz, 1H), 3.14 (dd, *J* = 18.3 Hz, *J* = 4.7 Hz, 1H); ^13^C NMR (100 MHz, DMSO-*d*_6_): *δ* 164.80, 156.86, 155.44, 154.42, 151.32, 136.53, 131.85, 131.68, 131.52, 130.36, 129.63, 128.54, 125.76, 121.61, 121.53, 121.44, 121.34, 113.95, 112.25, 69.51, 66.43, 54.58, 45.12; HRMS (AP-ESI) *m*/*z* [M+H]^+^ calcd for C_25_H_24_BrN_4_O_5_: 539.0930, found: 539.0938.

### 3.2. Biological Evaluation

#### 3.2.1. Enzymatic Inhibition Assay against APN In Vitro

The enzymatic inhibitory activities of target compounds were evaluated using *L*-Leu-*p*-nitroanilide (Sigma-Aldrich, St. Louis, MO, USA) as substrate and soluble APN from porcine kidney microsomal (Enzo Life Sciences, Farmingdale, NY, USA) as enzyme. Firstly, the substrate was dissolved in DMSO to a concentration of 16 mmol/L and the enzyme was dissolved in buffer solution of 50 mM PBS (pH = 7.2) for a concentration of 0.15 IU/L. Subsequently, inhibitors (40 μL) and PBS (145 μL) were added into the 96-well plates, followed by the addition of substrate (5 μL) and enzyme (10 μL). After incubation at 37 °C for 30 min, the absorbance values were measured at 405 nm with a plate reader (Varioskan, Thermo Fisher Scientific, Waltham, MA, USA).

#### 3.2.2. Anti-Proliferative Assay

The MTT method was used for the determination of anti-proliferative activities of selected compounds against tumor cells. Human histiocytic lymphoma cells U937, human chronic myeloid leukemia cells K562, liver carcinoma alexander cells PLC/PRF/5, human prostate cancer cells PC-3, human ovarian clear cell carcinoma cells ES-2, human hepatocellular carcinoma cells HepG2 were selected as tested tumor cell lines. Firstly, the cells were cultured in RPMI 1640 medium with 10% FBS at 37 °C in 5% CO_2_ humidified incubator. Cells (100 μL) in logarithmic phase were inoculated in 96-well plates and allowed to grow for 4 h. Subsequently, inhibitors (100 μL) at the tested concentration were added, followed by incubation for 48 h. Then, to the mixture was added MTT solution (20 μL, 5 mg/mL) and further incubated for 4 h. The plates were centrifuged at 800 rpm for 3 min. The supernatant was poured off and the formed formazan was dissolved in DMSO (200 μL). Finally, the mixture was shaken for 15 min and the absorbance values of the formazan solution were measured at 570 nm with a plate reader (Varioskan, Thermo, Waltham, MA, USA). All the tumor cells were owned by our laboratory.

#### 3.2.3. Rat Aortic Ring Assay

The thoracic aortas were separated from 8- to 10-week-old male Sprague Dawley rats and cut into rings with a 1-mm-long cross-section after removal of the connective tissues around the aortas. The fresh aortic ring was added into the 96-well plates pre-coated with matrigel (50 μL, BD bioscience), and covered with another 50 μL of matrigel. After incubation at 37 °C in 5% CO_2_ for 0.5 h, the tested compounds (100 μL) in M199 culture medium with 10% FBS was added to the mixture and incubated at 37 °C in 5% CO_2_ for 9 d. For the treatment group, the M199 culture medium with 10% FBS and inhibitors was changed every three days. For the control group, the M199 culture medium with 10% FBS and 0.5% DMSO was changed every three days. The formed micro-vessels were photographed by inverted microscope at 100× magnification. Experiments were repeated three times. All experiments involving laboratory animals were performed with the approval of the Laboratory Animal Welfare Review Committee of Shandong University of Traditional Chinese Medicine.

### 3.3. Molecular Docking Studies

In the APN-bestatin co-crystal structure (PDB code: 2DQM), the residues in a 10.0 Å radius circle around bestatin was selected as the active site. Compound **14o** was docked into it using Sybyl 2.1. Before the docking studies, the protein structure was prepared by deleting water molecules, adding hydrogen atoms, modifying atom types, and assigning with AMBER7 FF99 charges, followed by a 100-step minimization process. Using the Sybyl/Sketch module, the small molecular structures were prepared and further optimized using Powell’s method with the Tripos force field with convergence criterion set at 0.05 kcal/Å mol and assigned using the Gasteiger–Hückel method. Other docking parameters implied in the program were default values. Molecular docking was carried out via the Sybyl/Surflex-Dock module. The top-scoring pose was selected for discussions.

## 4. Conclusions

Summarily, one series of pyrazoline-based hydroxamate derivatives was designed, synthesized and evaluated as APNIs. Interestingly, in the enzymatic assay against APN, some compounds exhibited improved APN inhibitory activities relative to the lead compounds **13a**–**13c** and positive control bestatin. Among them, the 2,6-dichloro substituted compound **14o** (IC_50_ = 0.0062 ± 0.0004 μM) was the best one, with three orders of magnitude better APN inhibitory activity than bestatin. The SARs revealed that the substituents on the two phenyl groups of the diphenyl pyrazoline scaffold significantly impacted the capacities of inhibiting APN. Compounds with an *ortho* mono-substituent on the terminal phenyl group presented better APN inhibitory activities than their counterparts with a *meta* or *para* mono-substituent. Compounds with an *ortho*-substituent, such as 2-F (**14g**), 2-Cl (**14h**), 2-I (**14i**), 2-CH_3_ (**14k**), showed improved capacities of APN inhibition relative to the unsubstituted lead compound **13a**. However, introduction of substituents on another phenyl group resulted in decreased APN inhibitory activities. The putative interactions of **14o** with the active site of APN was predicted using Surflex-Dock module of Sybyl 2.1., which further supported our design strategy. Moreover, compared with bestatin, compound **14o** demonstrated superior anti-proliferative activities against tumor cells and anti-angiogenic activity, which confirmed the potency of **14o** as an anti-tumor lead.

## Figures and Tables

**Table 1:** The structures and IC_50_ values of target compounds against porcine kidney aminopeptidase N (APN).

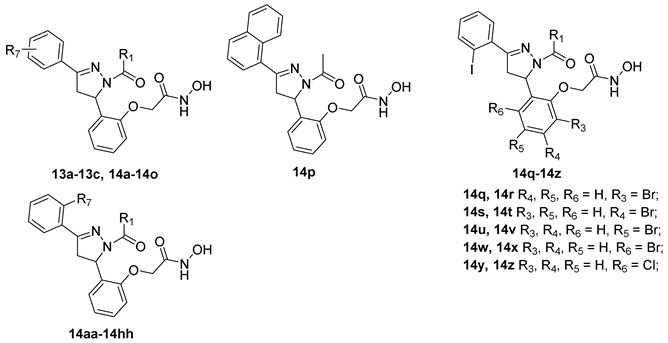							
Compd	R_1_	R_7_	APN IC_50_ (μM) ^a^	Compd	R_1_	R_7_	APN IC_50_ (μM) ^a^
**13a**	CH_3_	H	0.32 ± 0.01	**14q**	CH_3_	--	3.5 ± 0.2
**13b**	NH_2_	H	0.16 ± 0.01	**14r**	NH_2_	--	3.4 ± 0.3
**13c**	NH_2_	2,6-diCl	0.059 ± 0.002	**14s**	CH_3_	--	2.1 ± 0.2
**14a**	CH_3_	2-Br	0.046 ± 0.003	**14t**	NH_2_	--	1.6 ± 0.1
**14b**	CH_3_	3-Br	0.32 ± 0.03	**14u**	CH_3_	--	0.37 ± 0.02
**14c**	CH_3_	4-Br	4.4 ± 0.3	**14v**	NH_2_	--	0.54 ± 0.04
**14d**	CH_3_	2-OCH_3_	0.32 ± 0.01	**14w**	CH_3_	--	0.024 ± 0.001
**14e**	CH_3_	3-OCH_3_	0.37 ± 003	**14x**	NH_2_	--	0.090 ± 0.005
**14f**	CH_3_	4-OCH_3_	3.8 ± 0.2	**14y**	CH_3_	--	0.023 ± 0.001
**14g**	CH_3_	2-F	0.25 ± 0.01	**14z**	NH_2_	--	0.070 ± 0.003
**14h**	CH_3_	2-Cl	0.029 ± 0.002	**14aa**	CH_3_	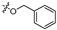	0.017 ± 0.001
**14i**	CH_3_	2-I	0.015 ± 0.001	**14bb**	NH_2_	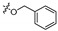	0.015 ± 0.001
**14j**	NH_2_	2-I	0.044 ± 0.003	**14cc**	CH_3_	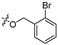	0.068 ± 0.004
**14k**	CH_3_	2-CH_3_	0.21 ± 0.01	**14dd**	NH_2_	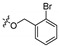	0.23 ± 0.01
**14l**	CH_3_	2,4-diCl	0.58 ± 0.04	**14ee**	CH_3_	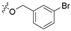	0.031 ± 0.002
**14m**	CH_3_	2,6-diOCH_3_	0.20 ± 0.01	**14ff**	NH_2_	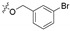	0.058 ± 0.003
**14n**	NH_2_	2,6-diOCH_3_	0.44 ± 0.03	**14gg**	CH_3_	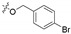	0.56 ± 0.04
**14o**	CH_3_	2,6-diCl	0.0062 ± 0.0004	**14hh**	NH_2_	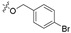	0.55 ± 0.04
**14p**	--	--	0.31 ± 0.02	Bestatin	--	--	8.8 ± 0.4

^a^ Assays were performed in triplicate; data are shown as mean ± SD.

**Table 2:** In vitro anti-proliferative activities of selected compounds against tumor cells.

Compound	IC_50_ (μM) ^a^					
	U937	K562	PLC/PRF/5	PC-3	ES-2	HepG2
**14o**	38.3 ± 3.6	10.4 ± 1.2	88.5 ± 7.4	45.8 ± 4.1	114.6 ± 9.7	84.2 ± 7.6
Bestatin	>500	>500	>500	>500	>500	>500

^a^ Assays were performed in triplicate; data are shown as mean ± SD.

## Data Availability

Not applicable.

## Supplementary Materials

The following supporting information can be downloaded at: https://www.mdpi.com/article/10.3390/molecules27238339/s1, the spectra data of some of intermediates **18**, **19** and **20**; the ^1^HNMR and ^13^C NMR spectra of target compounds **14h**–**14j**, **14o**, **14aa**–**14cc**, **14ee** and **14ff**.

## Sample Availability

Samples of the compounds **14i** and **14o** are available from the authors.
