# Supplementary material for: Development of Hydroxamate Derivatives Containing a Pyrazoline Moiety as APN Inhibitors to Overcome Angiogenesis
Source: Molecules. 2022 Nov 29;27(23):8339. doi: 10.3390/molecules27238339 (PMC9736874; doi:10.3390/molecules27238339)
Supplement: Supplementary file 1 [file molecules-27-08339-s001.zip › molecules-2031833-supplementary.pdf]

Supporting Information

S1. The spectra data of some of intermediates 18, 19 and 20.

*S1.1. Compounds **18i**, **18l**, **18o**, **18p** and **18r-18w** were prepared following the procedure described for the compound **18q**.*

(E)-3-(2-Hydroxyphenyl)-1-(2-iodophenyl)prop-2-en-1-one (**18i**)
Yellow solid, yield: 59%, mp: 110-112 °C. ESI-MS *m/z* 351.1 [M+H] $^+$.
(E)-1-(2,6-Dimethoxyphenyl)-3-(2-hydroxyphenyl)prop-2-en-1-one (**18l**)
Yellow solid, yield: 62%, mp: 152-154 °C. ESI-MS *m/z* 285.1 [M+H] $^+$.
(E)-3-(3-Bromo-2-hydroxyphenyl)-1-(2-iodophenyl)prop-2-en-1-one (**18o**)
Yellow solid, yield: 60%, mp: 108-110 °C. ESI-MS *m/z* 428.5 [M+H] $^+$.
(E)-3-(4-Bromo-2-hydroxyphenyl)-1-(2-iodophenyl)prop-2-en-1-one (**18p**)
Yellow solid, yield: 58%, mp: 162-164 °C. ESI-MS *m/z* 428.5 [M+H] $^+$.
(E)-3-(2-Bromo-6-hydroxyphenyl)-1-(2-iodophenyl)prop-2-en-1-one (**18r**)
Yellow solid, yield: 58%, mp: 166-168 °C. ESI-MS *m/z* 428.5 [M+H] $^+$.
(E)-3-(2-Chloro-6-hydroxyphenyl)-1-(2-iodophenyl)prop-2-en-1-one (**18s**)
Yellow solid, yield: 65%, mp: 154-156 °C. ESI-MS *m/z* 384.9 [M+H] $^+$.
(E)-1-(2-(Benzyloxy)phenyl)-3-(2-hydroxyphenyl)prop-2-en-1-one (**18t**)
Yellow solid, yield: 57%, mp: 146-148 °C. ESI-MS *m/z* 331.1 [M+H] $^+$.
(E)-1-(2-((2-Bromobenzyl)oxy)phenyl)-3-(2-hydroxyphenyl)prop-2-en-1-one (**18u**)
Yellow solid, yield: 63%, mp: 130-132 °C. ESI-MS *m/z* 409.2 [M+H] $^+$.
(E)-1-(2-((3-Bromobenzyl)oxy)phenyl)-3-(2-hydroxyphenyl)prop-2-en-1-one (**18v**)
Yellow solid, yield: 61%, mp: 134-136 °C. ESI-MS *m/z* 409.5 [M+H] $^+$.
(E)-1-(2-((4-Bromobenzyl)oxy)phenyl)-3-(2-hydroxyphenyl)prop-2-en-1-one (**18w**)
Yellow solid, yield: 57%, mp: 126-128 °C. ESI-MS *m/z* 409.5 [M+H] $^+$.

*S1.2. Compounds **19a-19c**, **19e-19i**, **19k-19m**, **19o-19q**, **19s**, **19u**, **19w**, **19y**, **19aa**, **19cc**, **19ee** and **19gg** were prepared following the procedure described for the compound **19d**.*

1-(3-(2-Bromophenyl)-5-(2-hydroxyphenyl)-4,5-dihydro-1H-pyrazol-1-yl) ethan-1-one (**19a**)
White solid, yield: 68%, mp: 166-168 °C. ESI-MS *m/z* 359.5 [M+H] $^+$.
1-(3-(3-Bromophenyl)-5-(2-hydroxyphenyl)-4,5-dihydro-1H-pyrazol-1-yl) ethan-1-one (**19b**)
White solid, yield: 70%, mp: 248-250 °C. ESI-MS *m/z* 359.5 [M+H] $^+$.
1-(3-(4-Bromophenyl)-5-(2-hydroxyphenyl)-4,5-dihydro-1H-pyrazol-1-yl) ethan-1-one (**19c**)
White solid, yield: 64%, mp: > 250 °C. ESI-MS *m/z* 359.5 [M+H] $^+$.
1-(5-(2-Hydroxyphenyl)-3-(3-methoxyphenyl)-4,5-dihydro-1H-pyrazol-1-yl) ethan-1-one (**19e**)
White solid, yield: 62%, mp: 204-208 °C. ESI-MS *m/z* 311.2 [M+H] $^+$.
1-(5-(2-Hydroxyphenyl)-3-(4-methoxyphenyl)-4,5-dihydro-1H-pyrazol-1-yl) ethan-1-one (**19f**)
White solid, yield: 60%, mp: 230-232 °C. ESI-MS *m/z* 311.2 [M+H] $^+$.
1-(3-(2-Fluorophenyl)-5-(2-hydroxyphenyl)-4,5-dihydro-1H-pyrazol-1-yl) ethan-1-one (**19g**)
White solid, yield: 53%, mp: 208-210 °C. ESI-MS *m/z* 299.1 [M+H] $^+$.
1-(3-(2-Chlorophenyl)-5-(2-hydroxyphenyl)-4,5-dihydro-1H-pyrazol-1-yl) ethan-1-one (**19h**)
White solid, yield: 59%, mp: 142-144 °C. ESI-MS *m/z* 315.2 [M+H] $^+$.
1-(5-(2-Hydroxyphenyl)-3-(2-iodophenyl)-4,5-dihydro-1H-pyrazol-1-yl) ethan-1-one (**19i**)

White solid, yield: 62%, mp: 152-154 °C. ESI-MS *m/z* 407.5 $[M+H]^+$.

1-(5-(2-Hydroxyphenyl)-3-(o-tolyl)-4,5-dihydro-1H-pyrazol-1-yl) ethan-1-one (**19k**)

White solid, yield: 64%, mp: 180-182 °C. ESI-MS *m/z* 295.2 $[M+H]^+$.

1-(3-(2,4-Dichlorophenyl)-5-(2-hydroxyphenyl)-4,5-dihydro-1H-pyrazol-1-yl) ethan-1-one (**19l**)

White solid, yield: 72%, mp: 226-228 °C. ESI-MS *m/z* 349.1 $[M+H]^+$.

1-(3-(2,6-Dimethoxyphenyl)-5-(2-hydroxyphenyl)-4,5-dihydro-1H-pyrazol-1-yl) ethan-1-one (**19m**)

White solid, yield: 69%, mp: 148-150 °C. ESI-MS *m/z* 341.4 $[M+H]^+$.

1-(3-(2,6-Dichlorophenyl)-5-(2-hydroxyphenyl)-4,5-dihydro-1H-pyrazol-1-yl) ethan-1-one (**19o**)

White solid, yield: 65%, mp: 180-182 °C. ESI-MS *m/z* 349.1 $[M+H]^+$.

1-(5-(2-Hydroxyphenyl)-3-(naphthalen-1-yl)-4,5-dihydro-1H-pyrazol-1-yl) ethan-1-one (**19p**)

White solid, yield: 71%, mp: 208-210 °C. ESI-MS *m/z* 331.2 $[M+H]^+$.

1-(5-(3-Bromo-2-hydroxyphenyl)-3-(2-iodophenyl)-4,5-dihydro-1H-pyrazol-1-yl) ethan-1-one (**19q**)

White solid, yield: 72%, mp: 132-134 °C. ESI-MS *m/z* 484.9 $[M+H]^+$.

1-(5-(4-Bromo-2-hydroxyphenyl)-3-(2-iodophenyl)-4,5-dihydro-1H-pyrazol-1-yl) ethan-1-one (**19s**)

White solid, yield: 74%, mp: 98-100 °C. ESI-MS *m/z* 484.9 $[M+H]^+$.

1-(5-(5-Bromo-2-hydroxyphenyl)-3-(2-iodophenyl)-4,5-dihydro-1H-pyrazol-1-yl) ethan-1-one (**19u**)

White solid, yield: 77%, mp: 176-178 °C. ESI-MS *m/z* 484.9 $[M+H]^+$.

1-(5-(2-Bromo-6-hydroxyphenyl)-3-(2-iodophenyl)-4,5-dihydro-1H-pyrazol-1-yl) ethan-1-one (**19w**)

White solid, yield: 68%, mp: 214-216 °C. ESI-MS *m/z* 484.9 $[M+H]^+$.

1-(5-(2-Chloro-6-hydroxyphenyl)-3-(2-iodophenyl)-4,5-dihydro-1H-pyrazol-1-yl) ethan-1-one (**19y**)

White solid, yield: 67%, mp: 204-206 ℃. ESI-MS *m/z* 440.9 $[M+H]^+$.

1-(3-(2-(Benzyloxy)phenyl)-5-(2-hydroxyphenyl)-4,5-dihydro-1H-pyrazol-1-yl) ethan-1-one (**19aa**)

White solid, yield: 70%, mp: 170-172 °C. ESI-MS *m/z* 387.5 $[M+H]^+$.

1-(3-(2-((2-Bromobenzyl)oxy)phenyl)-5-(2-hydroxyphenyl)-4,5-dihydro-1H-pyrazol-1-yl) ethan-1-one (**19cc**)

White solid, yield: 72%, mp: 176-178 °C. ESI-MS *m/z* 465.1 $[M+H]^+$.

1-(3-(2-((3-Bromobenzyl)oxy)phenyl)-5-(2-hydroxyphenyl)-4,5-dihydro-1H-pyrazol-1-yl) ethan-1-one (**19ee**)

White solid, yield: 67%, mp: 182-184 °C. ESI-MS *m/z* 465.1 $[M+H]^+$.

1-(3-(2-((4-Bromobenzyl)oxy)phenyl)-5-(2-hydroxyphenyl)-4,5-dihydro-1H-pyrazol-1-yl) ethan-1-one (**19gg**)

White solid, yield: 71%, mp: 142-144 °C. ESI-MS *m/z* 465.1 $[M+H]^+$.

*S1.3. Compounds **19n**, **19r**, **19t**, **19v**, **19x**, **19z**, **19bb**, **19dd**, **19ff** and **19hh** were prepared following the procedure described for the compound **19j**.*

3-(2,6-Dimethoxyphenyl)-5-(2-hydroxyphenyl)-4,5-dihydro-1H-pyrazole-1-carboxamide (**19n**)

White solid, yield: 64%, mp: 216-218 °C. ESI-MS *m/z* 342.5 [M+H] $^+$.

5-(3-Bromo-2-hydroxyphenyl)-3-(2-iodophenyl)-4,5-dihydro-1H-pyrazole-1-carboxamide (**19r**)

White solid, yield: 61%, mp: 184-186 °C. ESI-MS *m/z* 485.9 [M+H] $^+$.

5-(4-Bromo-2-hydroxyphenyl)-3-(2-iodophenyl)-4,5-dihydro-1H-pyrazole-1-carboxamide (**19t**)

White solid, yield: 65%, mp: 206-208 °C. ESI-MS *m/z* 485.9 [M+H] $^+$.

5-(5-Bromo-2-hydroxyphenyl)-3-(2-iodophenyl)-4,5-dihydro-1H-pyrazole-1-carboxamide (**19v**)

White solid, yield: 68%, mp: 220-222 °C. ESI-MS *m/z* 485.9 [M+H] $^+$.

5-(2-Bromo-6-hydroxyphenyl)-3-(2-iodophenyl)-4,5-dihydro-1H-pyrazole-1-carboxamide (**19x**)

White solid, yield: 65%, mp: 218-220 °C. ESI-MS *m/z* 485.9 [M+H] $^+$.

5-(2-Chloro-6-hydroxyphenyl)-3-(2-iodophenyl)-4,5-dihydro-1H-pyrazole-1-carboxamide (**19z**)

White solid, yield: 66%, mp: 218-220 °C. ESI-MS *m/z* 441.9 [M+H] $^+$.

3-(2-(Benzyloxy)phenyl)-5-(2-hydroxyphenyl)-4,5-dihydro-1H-pyrazole-1-carboxamide (**19bb**)

White solid, yield: 62%, mp: 172-174 °C. ESI-MS *m/z* 388.2 [M+H] $^+$.

3-(2-((2-Bromobenzyl)oxy)phenyl)-5-(2-hydroxyphenyl)-4,5-dihydro-1H-pyrazole-1-carboxamide (**19dd**)

White solid, yield: 63%, mp: 132-134 °C. ESI-MS *m/z* 466.1 [M+H] $^+$.

3-(2-((3-Bromobenzyl)oxy)phenyl)-5-(2-hydroxyphenyl)-4,5-dihydro-1H-pyrazole-1-carboxamide (**19ff**)

White solid, yield: 60%, mp: 200-202 °C. ESI-MS *m/z* 466.1 [M+H] $^+$.

3-(2-((4-Bromobenzyl)oxy)phenyl)-5-(2-hydroxyphenyl)-4,5-dihydro-1H-pyrazole-1-carboxamide (**19hh**)

White solid, yield: 64%, mp: 174-176 °C. ESI-MS *m/z* 466.1 [M+H] $^+$.

*S1.4. Compounds **20a-20c**, **20e-20z** and **20aa-20hh** were prepared following the procedure described for the compound **20d**.*

Methyl 2-(2-(1-acetyl-3-(2-bromophenyl)-4,5-dihydro-1H-pyrazol-5-yl) phenoxy) acetate (**20a**)

White solid, yield: 64%, mp: 92-94 °C. ESI-MS *m/z* 431.5 [M+H] $^+$.

Methyl 2-(2-(1-acetyl-3-(3-bromophenyl)-4,5-dihydro-1H-pyrazol-5-yl) phenoxy) acetate (**20b**)

White solid, yield: 61%, mp: 142-144 °C. ESI-MS *m/z* 431.5 [M+H] $^+$.

Methyl 2-(2-(1-acetyl-3-(4-bromophenyl)-4,5-dihydro-1H-pyrazol-5-yl) phenoxy) acetate (**20c**)

White solid, yield: 68%, mp: 158-160 °C. ESI-MS *m/z* 431.5 [M+H] $^+$.

Methyl 2-(2-(1-acetyl-3-(3-methoxyphenyl)-4,5-dihydro-1H-pyrazol-5-yl) phenoxy) acetate (**20e**)

White solid, yield: 59%, mp: 138-140 °C. ESI-MS *m/z* 383.5 [M+H] $^+$.

Methyl 2-(2-(1-acetyl-3-(4-methoxyphenyl)-4,5-dihydro-1H-pyrazol-5-yl) phenoxy) acetate (**20f**)

White solid, yield: 67%, mp: 106-108 °C. ESI-MS *m/z* 383.5 [M+H] $^+$.

Methyl 2-(2-(1-acetyl-3-(2-fluorophenyl)-4,5-dihydro-1H-pyrazol-5-yl) phenoxy) acetate (**20g**)

White solid, yield: 68%, mp: 110-112 °C. ESI-MS *m/z* 371.3 [M+H] $^+$.

Methyl 2-(2-(1-acetyl-3-(2-chlorophenyl)-4,5-dihydro-1H-pyrazol-5-yl)phenoxy) acetate (**20h**)

White solid, yield: 70%, mp: 108-110 °C. ESI-MS *m/z* 413.5 [M+H] $^+$.

Methyl 2-(2-(1-acetyl-3-(2-iodophenyl)-4,5-dihydro-1H-pyrazol-5-yl)phenoxy) acetate (**20i**)

White solid, yield: 62%, mp: 98-100 °C. ESI-MS *m/z* 479.2 [M+H] $^+$.

Methyl 2-(2-(1-carbamoyl-3-(2-iodophenyl)-4,5-dihydro-1H-pyrazol-5-yl)phenoxy)acetate (**20j**)

White solid, yield: 68%, mp: 184-186 °C. ESI-MS *m/z* 480.5 [M+H] $^+$.

Methyl 2-(2-(1-acetyl-3-(o-tolyl)-4,5-dihydro-1H-pyrazol-5-yl)phenoxy) acetate (**20k**)

White solid, yield: 62%, mp: 122-124 °C. ESI-MS *m/z* 367.1 [M+H] $^+$.

Methyl 2-(2-(1-acetyl-3-(2,4-dichlorophenyl)-4,5-dihydro-1H-pyrazol-5-yl) phenoxy) acetate (**20l**)

White solid, yield: 65%, mp: 96-98 °C. ESI-MS *m/z* 421.1 [M+H] $^+$.

Methyl 2-(2-(1-acetyl-3-(2,6-dimethoxyphenyl)-4,5-dihydro-1H-pyrazol-5-yl)phenoxy) acetate (**20m**)

White solid, yield: 60%, mp: 164-166 °C. ESI-MS *m/z* 413.5 [M+H] $^+$.

Methyl 2-(2-(1-carbamoyl-3-(2,6-dimethoxyphenyl)-4,5-dihydro-1H-pyrazol-5-yl)phenoxy) acetate (**20n**)

White solid, yield: 69%, mp: 170-172 °C. ESI-MS *m/z* 414.5 [M+H] $^+$.

Methyl 2-(2-(1-acetyl-3-(2,6-dichlorophenyl)-4,5-dihydro-1H-pyrazol-5-yl)phenoxy) acetate (**20o**)

White solid, yield: 61%, mp: 162-164 °C. ESI-MS *m/z* 421.3 [M+H] $^+$.

Methyl 2-(2-(1-acetyl-3-(naphthalen-1-yl)-4,5-dihydro-1H-pyrazol-5-yl)phenoxy) acetate (**20p**)

White solid, yield: 67%, mp: 170-172 °C. ESI-MS *m/z* 403.1 [M+H] $^+$.

Methyl 2-(2-(1-acetyl-3-(2-iodophenyl)-4,5-dihydro-1H-pyrazol-5-yl)-6-bromophenoxy) acetate (**20q**)

White solid, yield: 62%, mp: 110-112 °C. ESI-MS *m/z* 556.9 [M+H] $^+$.

Methyl 2-(2-bromo-6-(1-carbamoyl-3-(2-iodophenyl)-4,5-dihydro-1H-pyrazol-5-yl)phenoxy) acetate (**20r**)

White solid, yield: 64%, mp: 166-168 °C. ESI-MS *m/z* 558.0 [M+H] $^+$.

Methyl 2-(2-(1-acetyl-3-(2-iodophenyl)-4,5-dihydro-1H-pyrazol-5-yl)-5- bromophenoxy) acetate (**20s**)

White solid, yield: 66%, mp: 114-116 °C. ESI-MS *m/z* 556.9 [M+H] $^+$.

Methyl 2-(5-bromo-2-(1-carbamoyl-3-(2-iodophenyl)-4,5-dihydro-1H-pyrazol-5-yl)phenoxy) acetate (**20t**)

White solid, yield: 72%, mp: 148-150 °C. ESI-MS *m/z* 557.9 [M+H] $^+$.

Methyl 2-(2-(1-acetyl-3-(2-iodophenyl)-4,5-dihydro-1H-pyrazol-5-yl)-4-bromophenoxy) acetate (**20u**)

White solid, yield: 70%, mp: 82-84 °C. ESI-MS *m/z* 556.9 [M+H] $^+$.

Methyl 2-(4-bromo-2-(1-carbamoyl-3-(2-iodophenyl)-4,5-dihydro-1H-pyrazol-5-yl)phenoxy) acetate (**20v**)

White solid, yield: 65%, mp: 192-194 °C. ESI-MS *m/z* 557.9 [M+H] $^+$.

Methyl 2-(2-(1-acetyl-3-(2-iodophenyl)-4,5-dihydro-1H-pyrazol-5-yl)-3-bromophenoxy)acetate

(**20w**)

White solid, yield: 63%, mp: 112-114 °C. ESI-MS *m/z* 556.9 [M+H] $^{+}$.

Methyl 2-(3-bromo-2-(1-carbamoyl-3-(2-iodophenyl)-4,5-dihydro-1H-pyrazol-5-yl)phenoxy) acetate (**20x**)

White solid, yield: 61%, mp: 188-190 °C. ESI-MS *m/z* 557.9 [M+H] $^{+}$.

Methyl 2-(2-(1-acetyl-3-(2-iodophenyl)-4,5-dihydro-1H-pyrazol-5-yl)-3-chlorophenoxy)acetate (**20y**)

White solid, yield: 69%, mp: 118-120 °C. ESI-MS *m/z* 513.2 [M+H] $^{+}$.

Methyl 2-(2-(1-carbamoyl-3-(2-iodophenyl)-4,5-dihydro-1H-pyrazol-5-yl)-3-chlorophenoxy) acetate (**20z**)

White solid, yield: 68%, mp: 206-208 °C. ESI-MS *m/z* 514.1 [M+H] $^{+}$.

Methyl 2-(2-(1-acetyl-3-(2-(benzyloxy)phenyl)-4,5-dihydro-1H-pyrazol-5-yl)phenoxy) acetate (**20aa**)

White solid, yield: 58%, mp: 130-132 °C. ESI-MS *m/z* 459.1 [M+H] $^{+}$.

Methyl 2-(2-(3-(2-(benzyloxy)phenyl)-1-carbamoyl-4,5-dihydro-1H-pyrazol-5-yl)phenoxy) acetate (**20bb**)

White solid, yield: 61%, mp: 178-180 °C. ESI-MS *m/z* 460.2 [M+H] $^{+}$.

Methyl2-(2-(1-acetyl-3-(2-((2-bromobenzyl)oxy)phenyl)-4,5-dihydro-1H-pyrazol-5-yl) phenoxy)acetate (**20cc**)

White solid, yield: 65%, mp: 120-122 °C. ESI-MS *m/z* 537.2 [M+H] $^{+}$.

Methyl 2-(2-(3-(2-((2-bromobenzyl)oxy)phenyl)-1-carbamoyl-4,5-dihydro-1H-pyrazol-5-yl)phenoxy)acetate (**20dd**)

White solid, yield: 67%, mp: 152-154 °C. ESI-MS *m/z* 538.5 [M+H] $^{+}$.

Methyl 2-(2-(1-acetyl-3-(2-((3-bromobenzyl)oxy)phenyl)-4,5-dihydro-1H-pyrazol-5-yl) phenoxy) acetate (**20ee**)

White solid, yield: 68%, mp: 102-104 °C. ESI-MS *m/z* 537.2 [M+H] $^{+}$.

Methyl 2-(2-(3-(2-((3-bromobenzyl)oxy)phenyl)-1-carbamoyl-4,5-dihydro-1H-pyrazol-5-yl) phenoxy)acetate (**20ff**)

White solid, yield: 70%, mp: 128-130 ℃. ESI-MS *m/z* 538.5 [M+H] $^{+}$.

Methyl 2-(2-(1-acetyl-3-(2-((4-bromobenzyl)oxy)phenyl)-4,5-dihydro-1H-pyrazol-5-yl) phenoxy)acetate (**20gg**)

White solid, yield: 71%, mp: 106-108 °C. ESI-MS *m/z* 537.2 [M+H] $^{+}$.

Methyl 2-(2-(3-(2-((4-bromobenzyl)oxy)phenyl)-1-carbamoyl-4,5-dihydro-1H-pyrazol-5-yl) phenoxy)acetate (**20hh**)

White solid, yield: 67%, mp: 134-136 °C. ESI-MS *m/z* 538.5 [M+H] $^{+}$.

S2. The ^{1}H NMR and ^{13}C NMR spectra of target compounds 14h-14j, 14o, 14aa-14cc, 14ee, and 14ff.

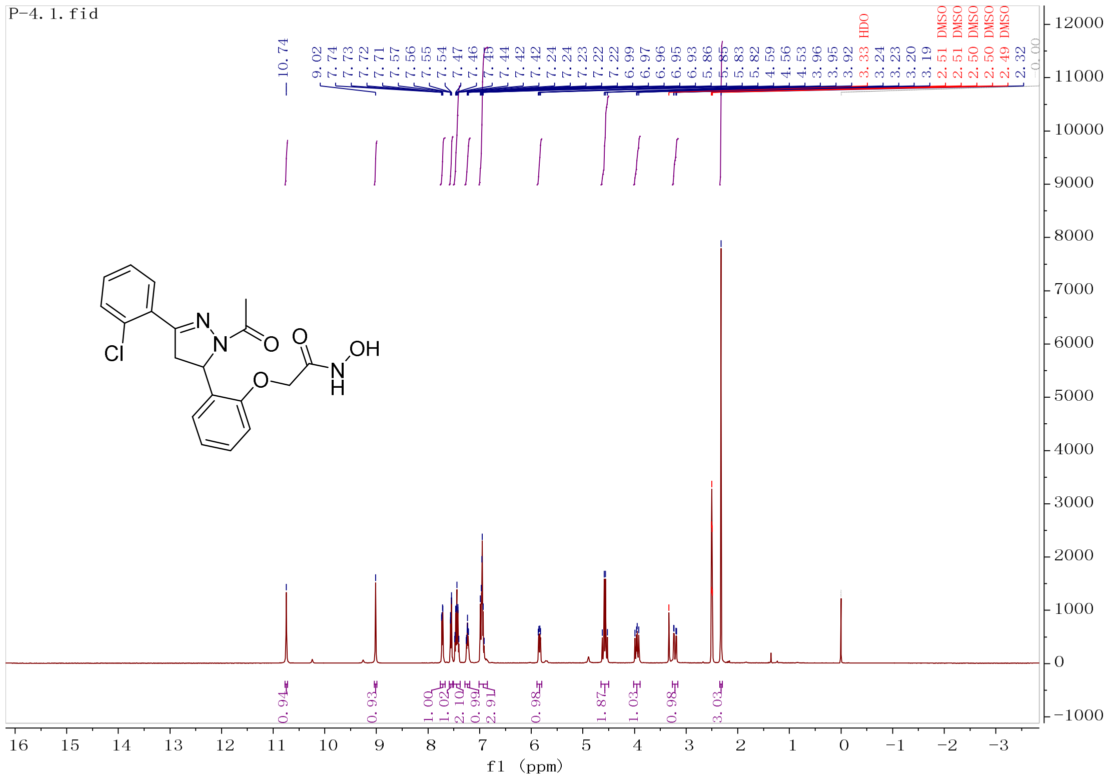

^{1}H NMR spectrum of compound **14h**

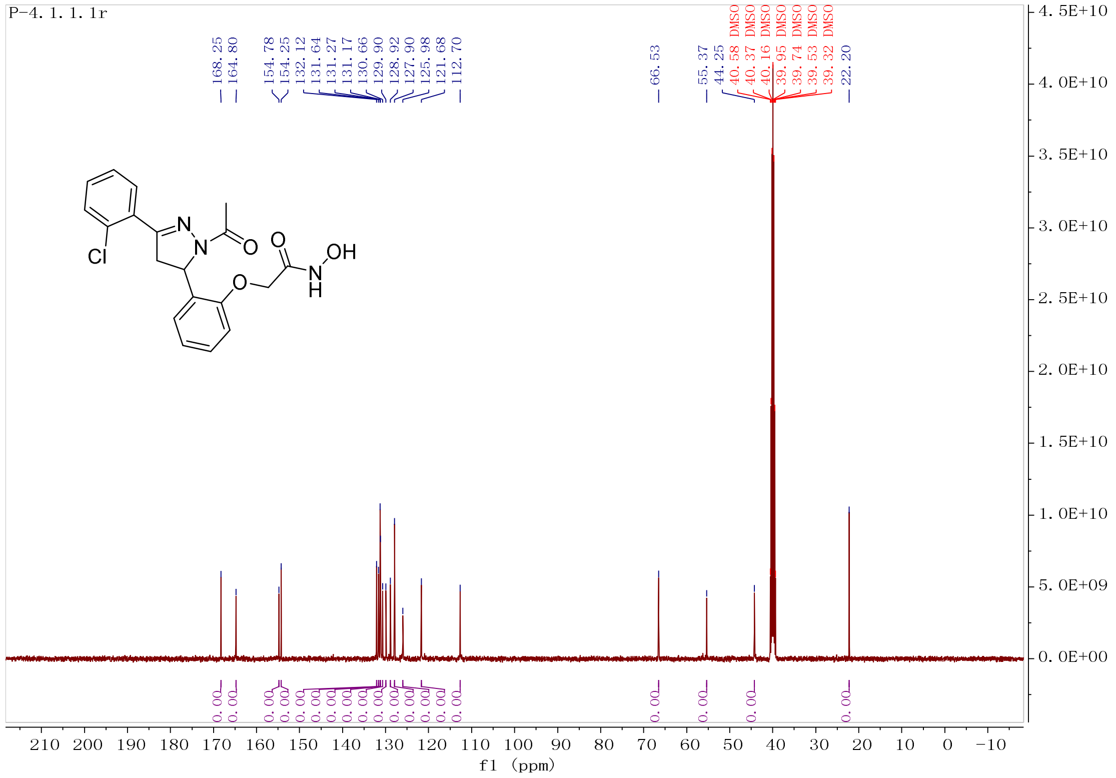

^{13}C NMR spectrum of compound **14h**

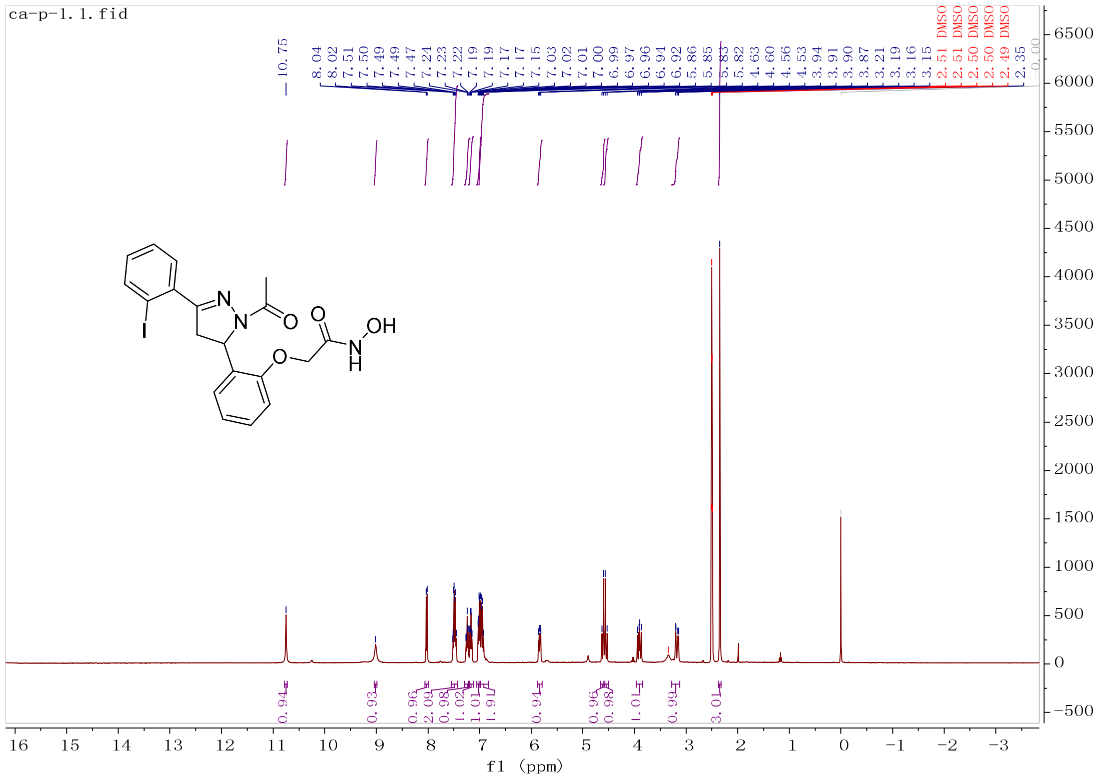

^{1}H NMR spectrum of compound **14i**

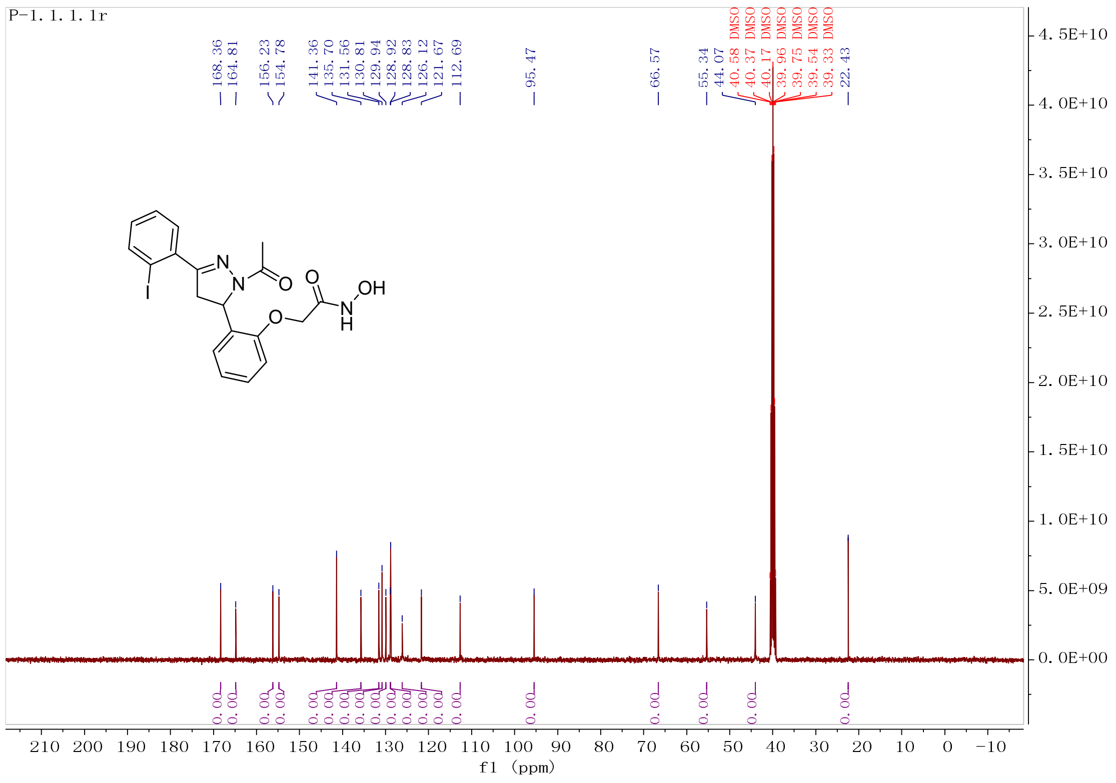

^{13}C NMR spectrum of compound **14i**

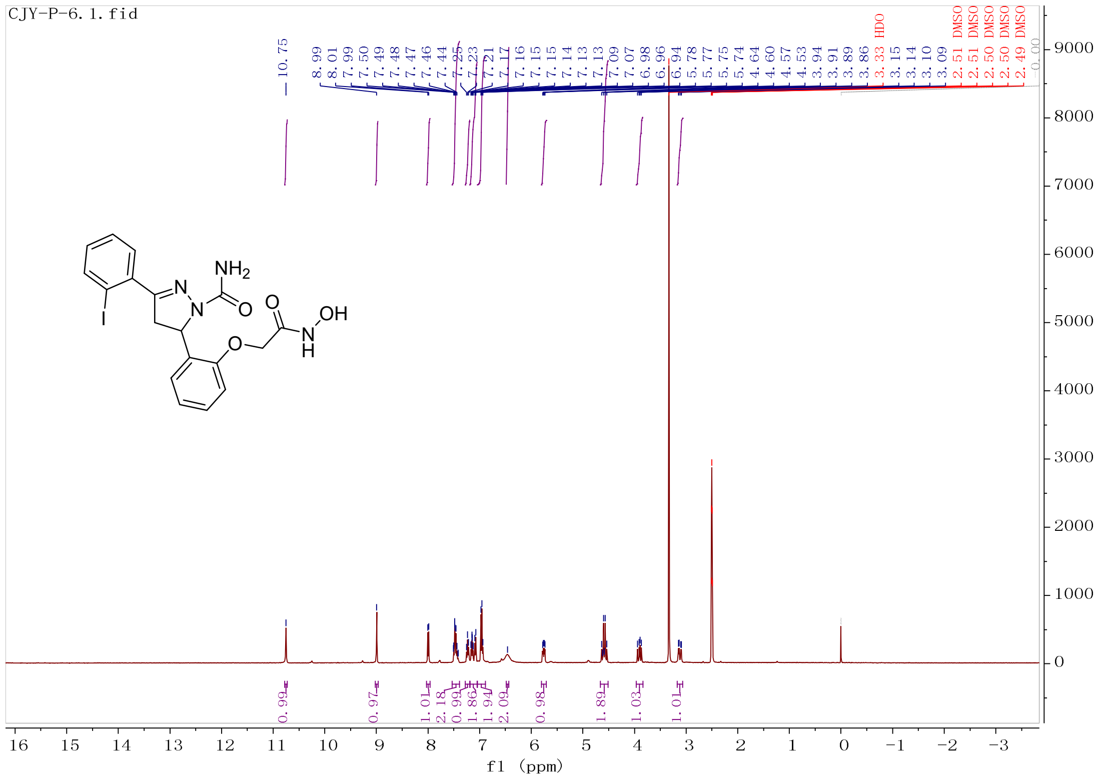

^{1}H NMR spectrum of compound **14j**

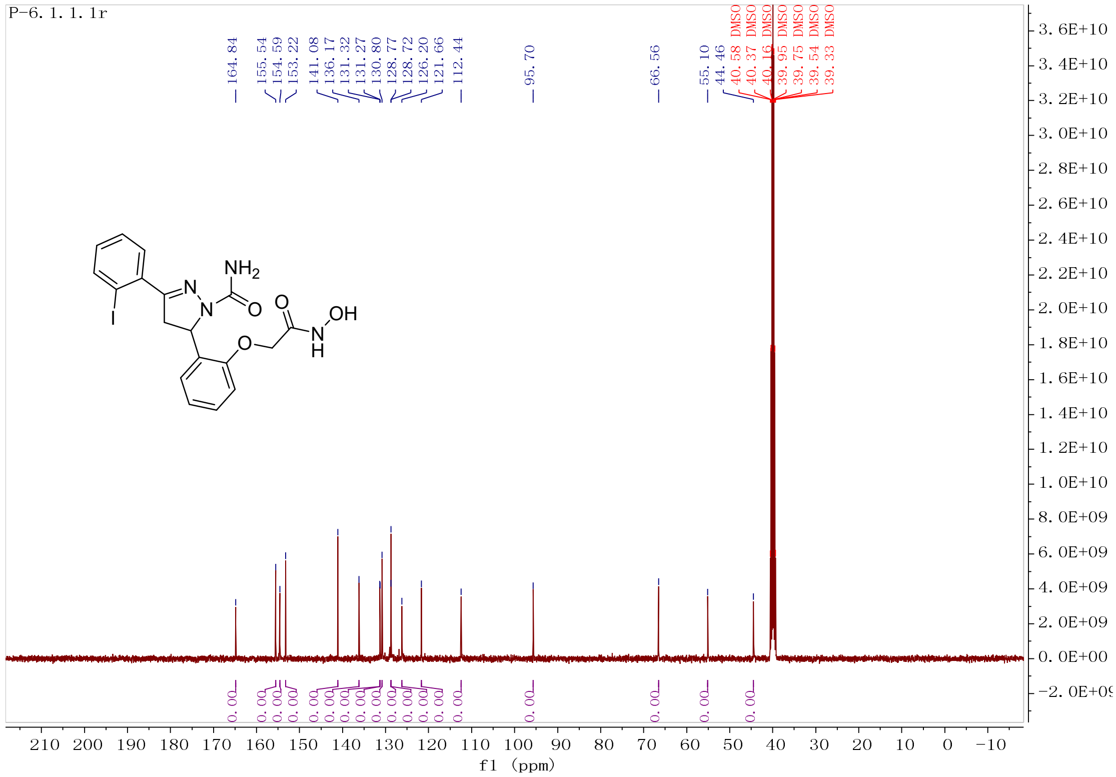

^{13}C NMR spectrum of compound **14j**

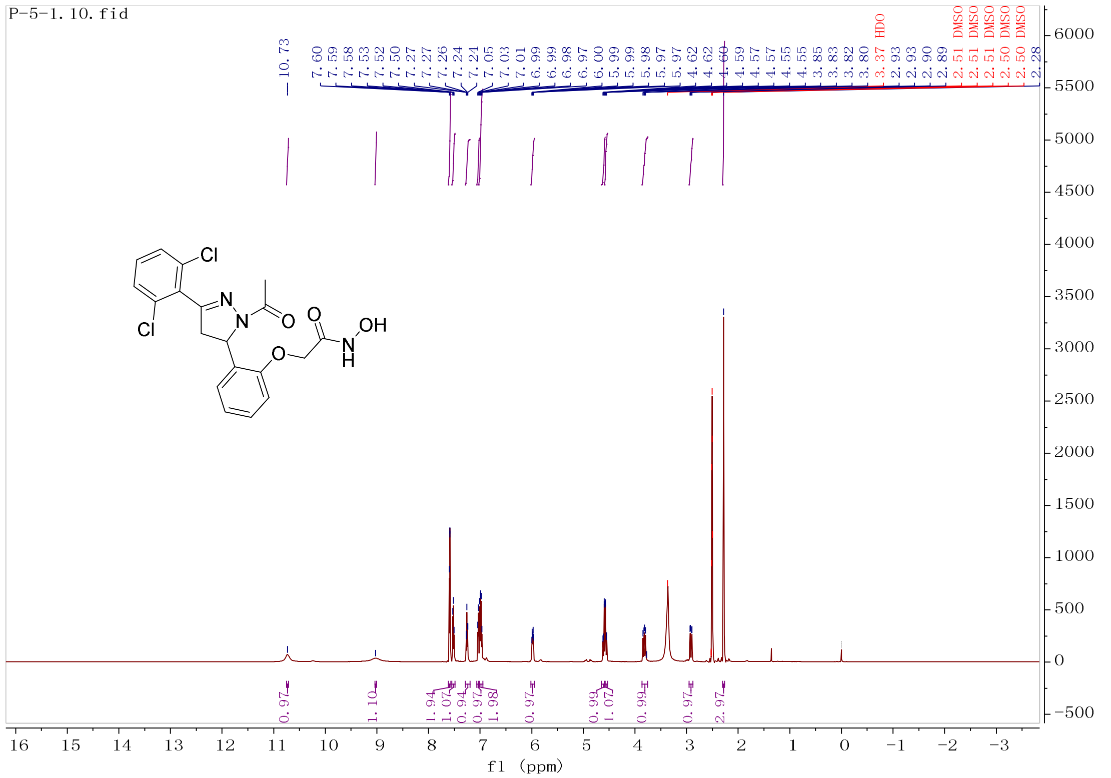

^{1}H NMR spectrum of compound **14o**

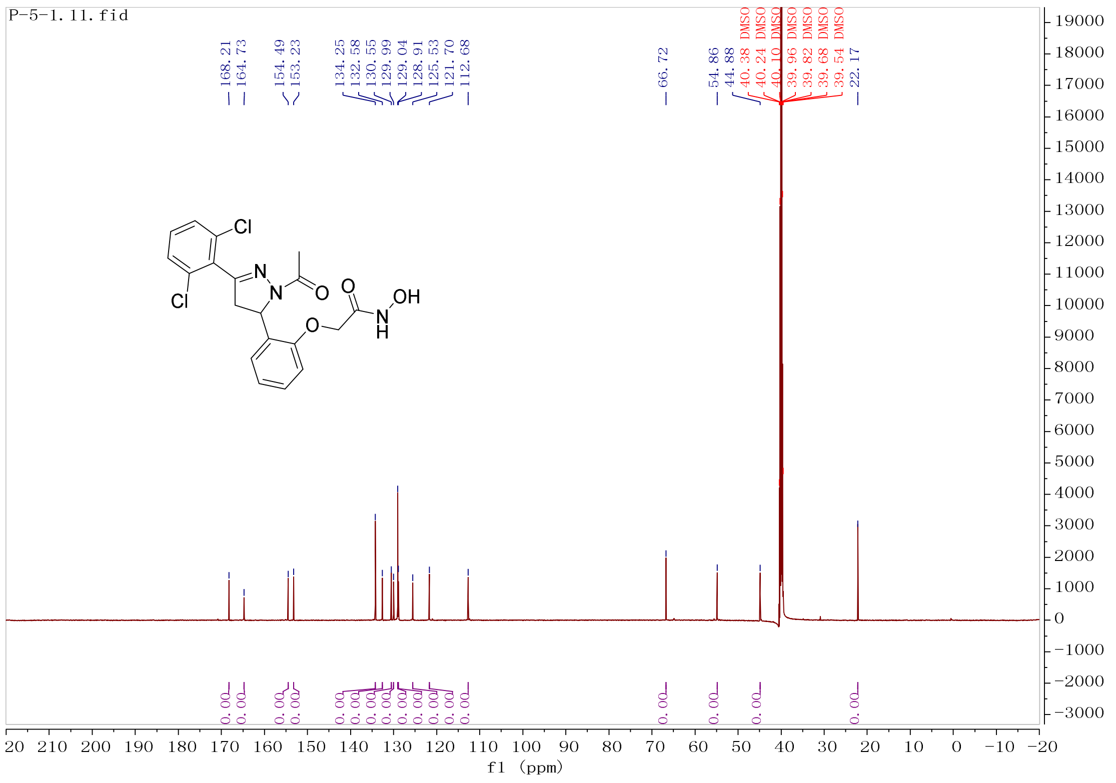

^{13}C NMR spectrum of compound **14o**

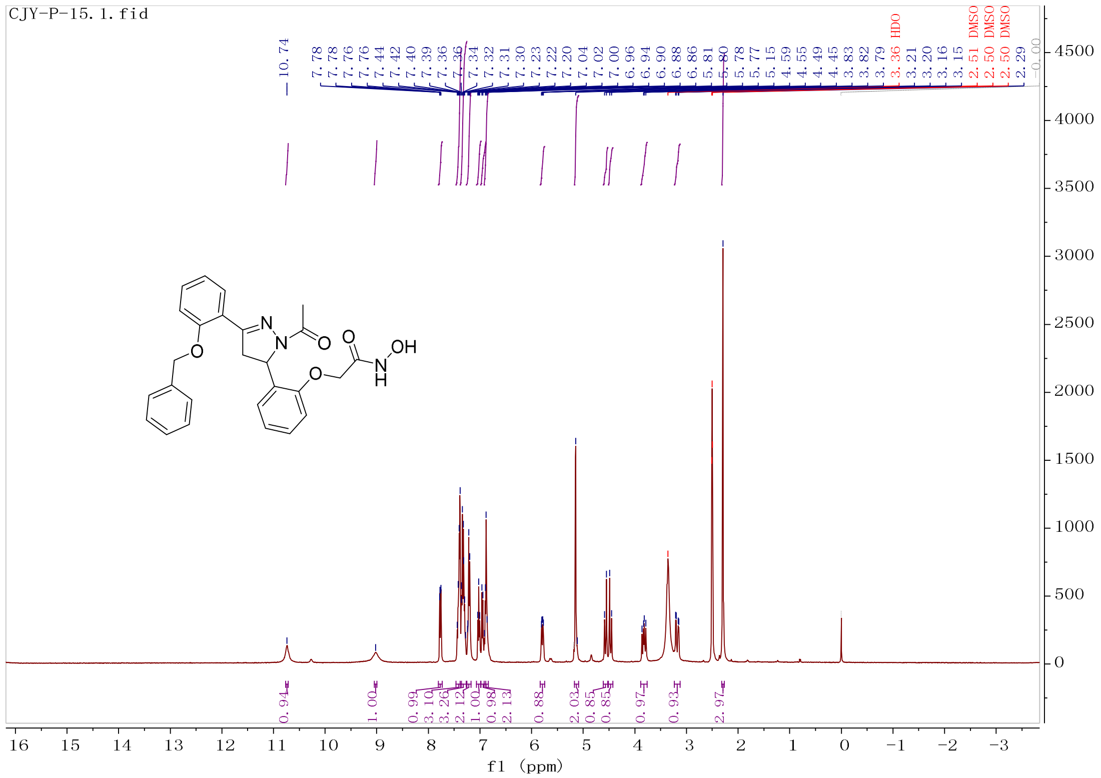

^{1}H NMR spectrum of compound **14aa**

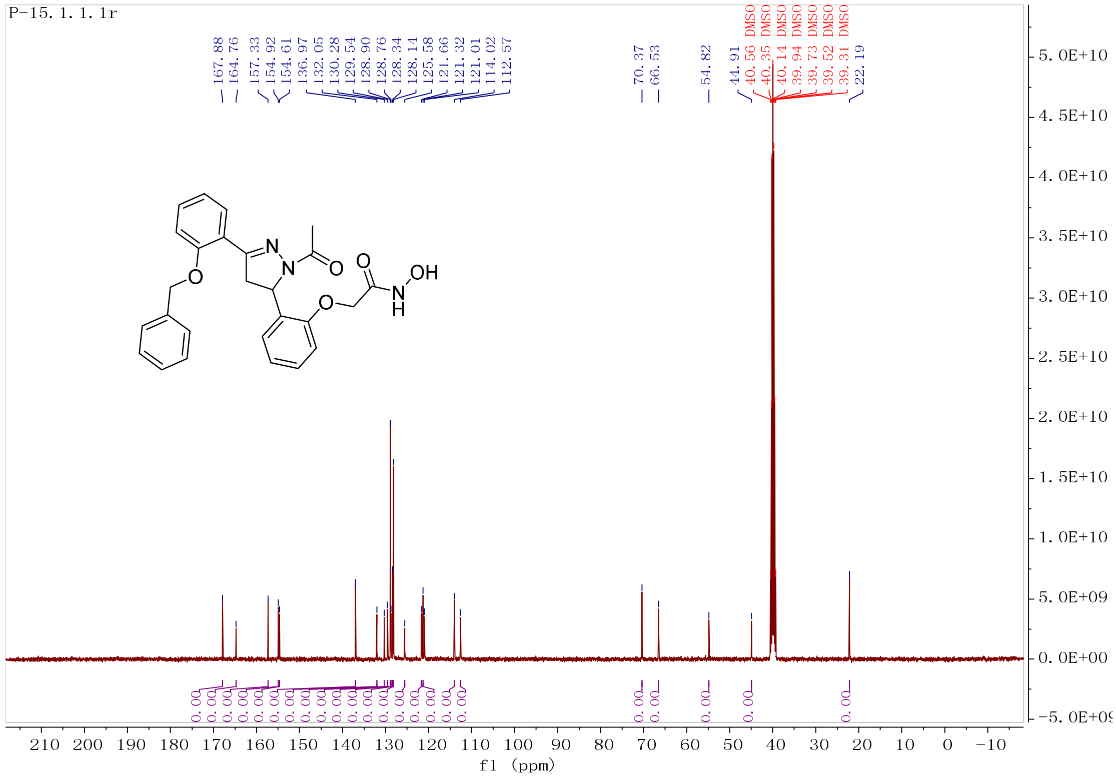

^{13}C NMR spectrum of compound **14aa**

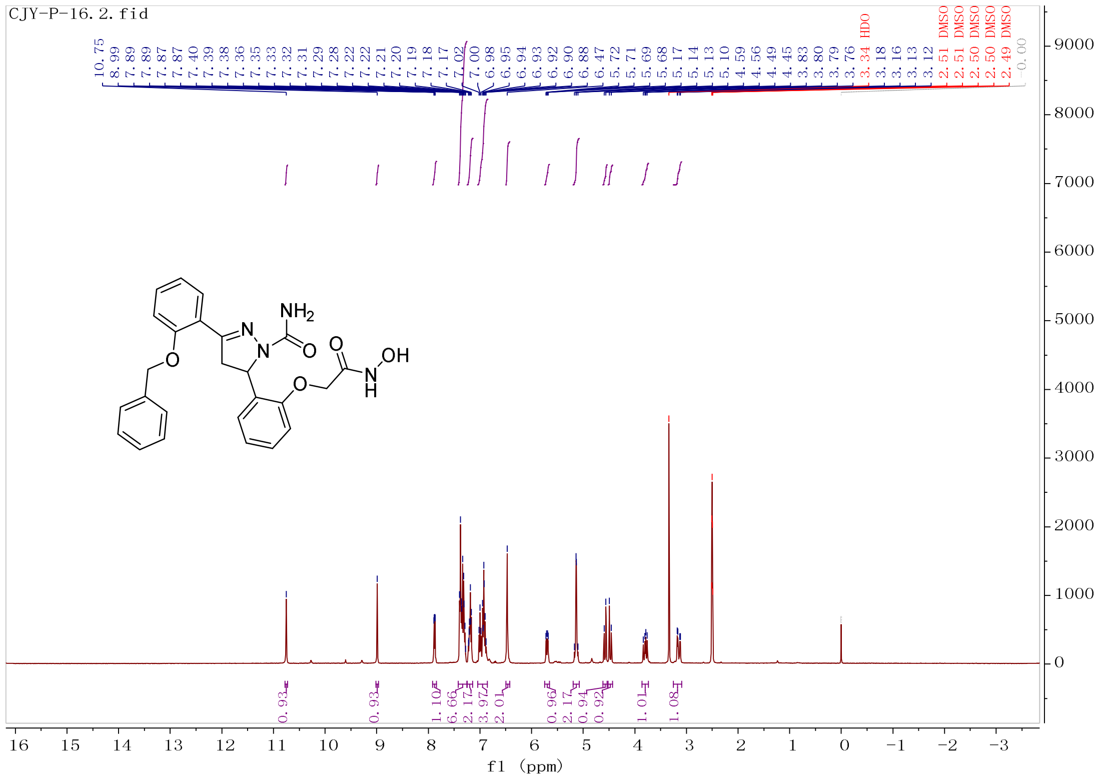

^{1}H NMR spectrum of compound **14bb**

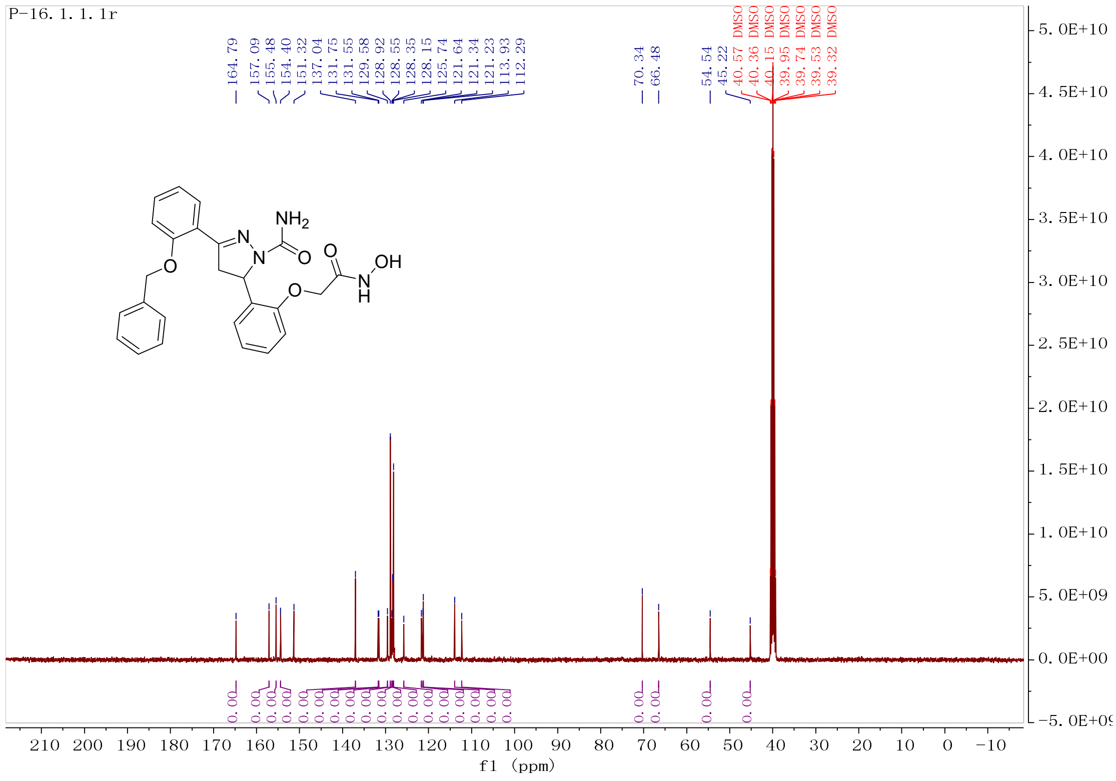

^{13}C NMR spectrum of compound **14bb**

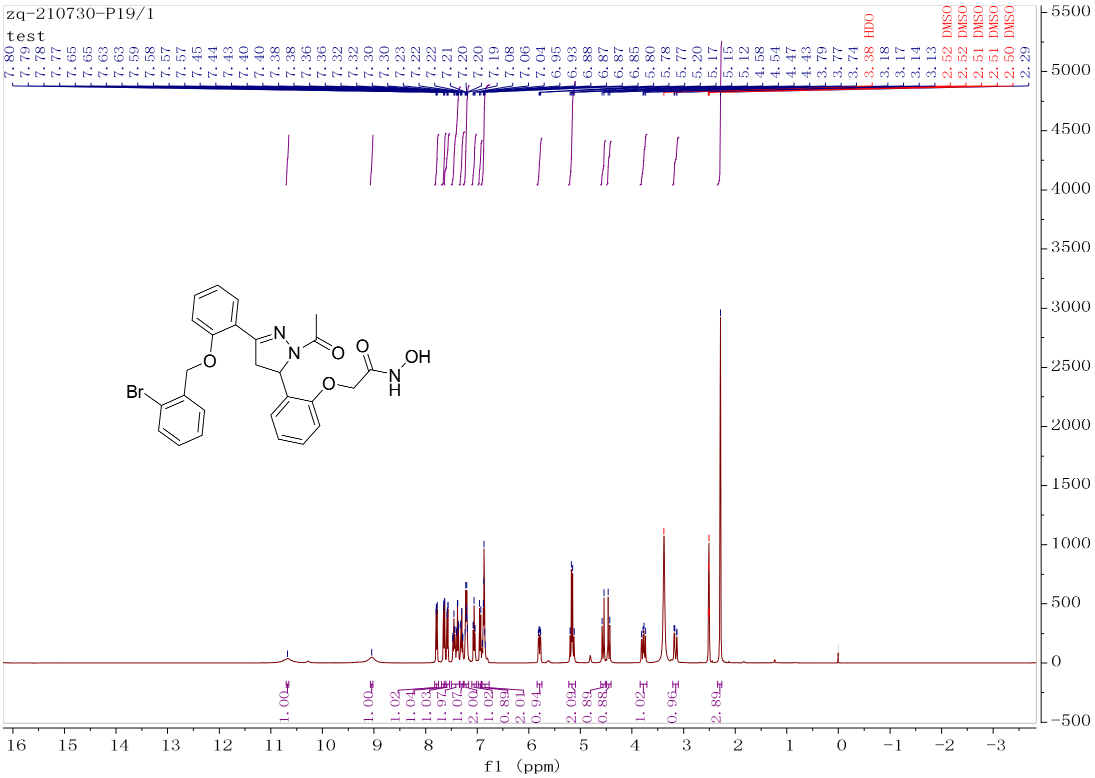

^{1}H NMR spectrum of compound **14cc**

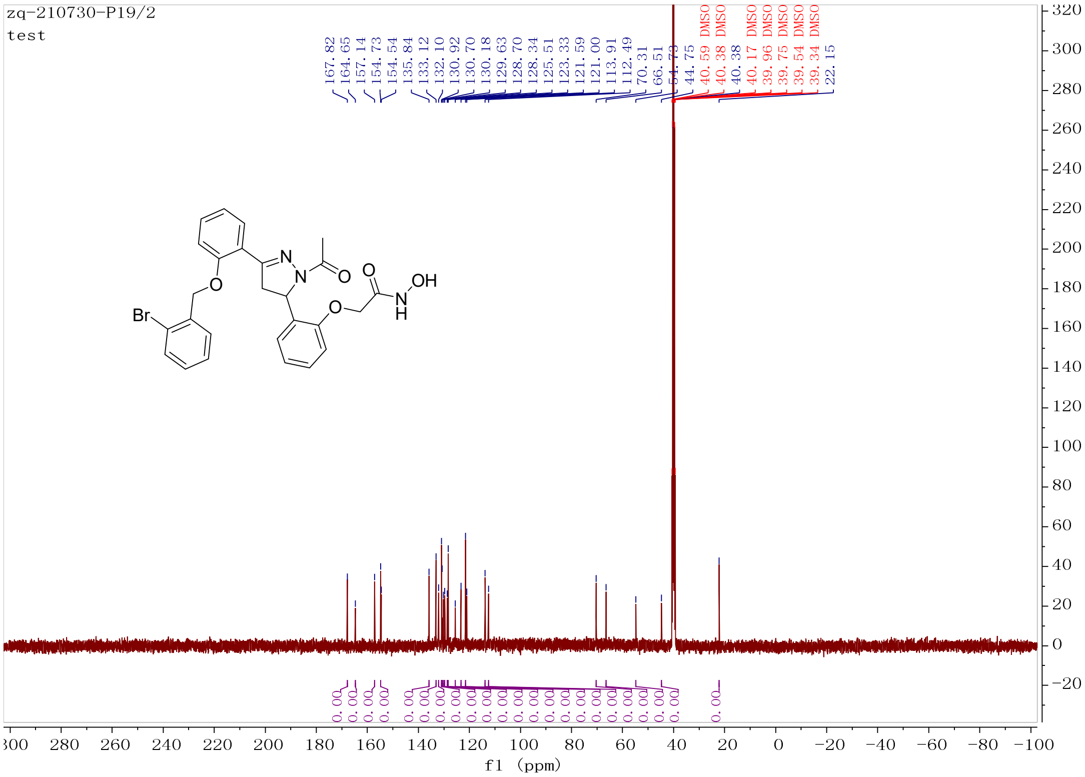

^{13}C NMR spectrum of compound **14cc**

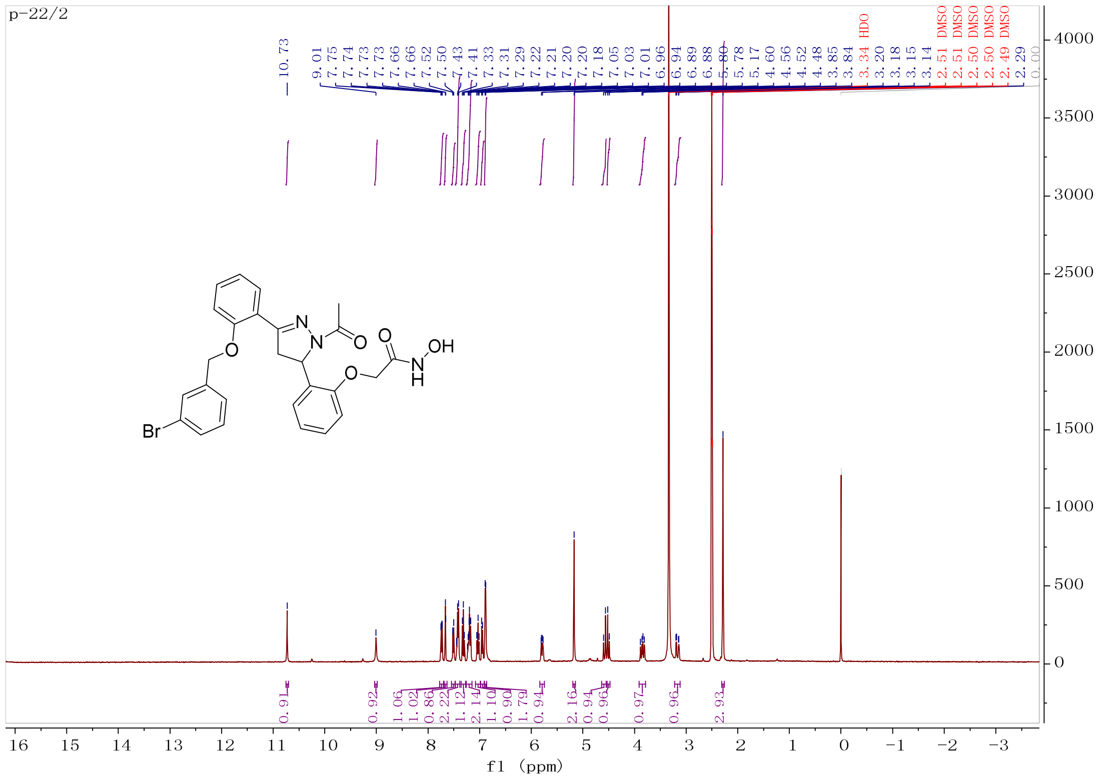

^{1}H NMR spectrum of compound **14ee**

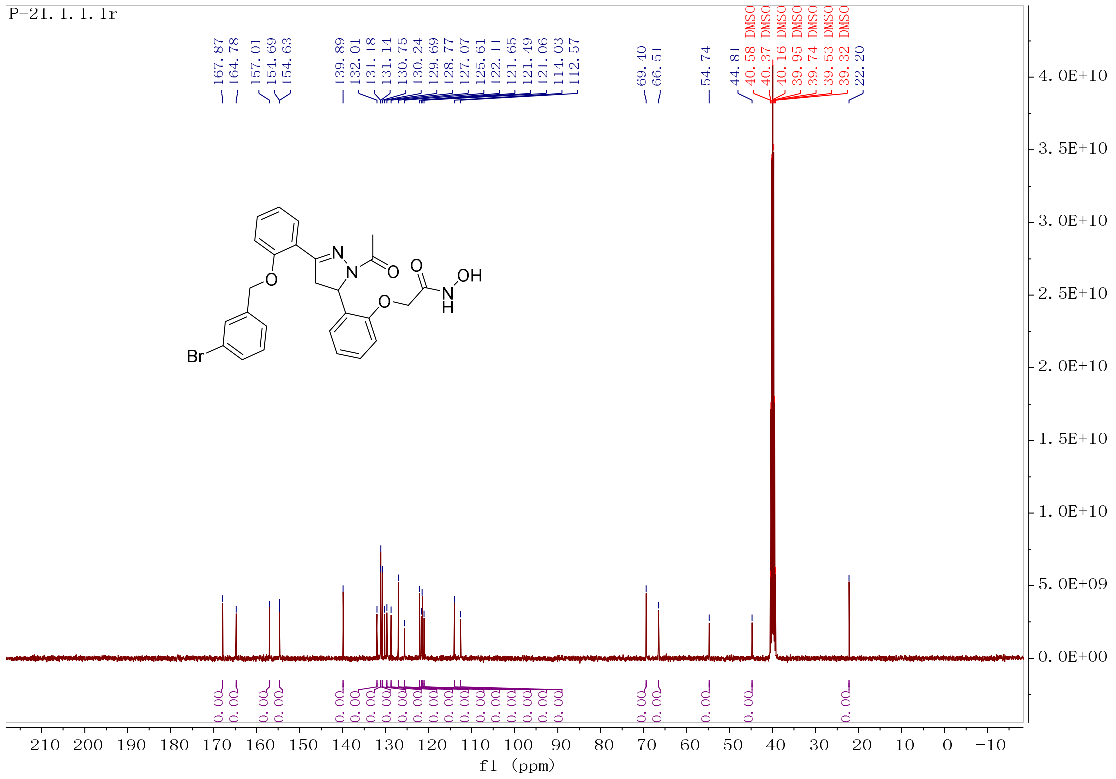

^{13}C NMR spectrum of compound **14ee**

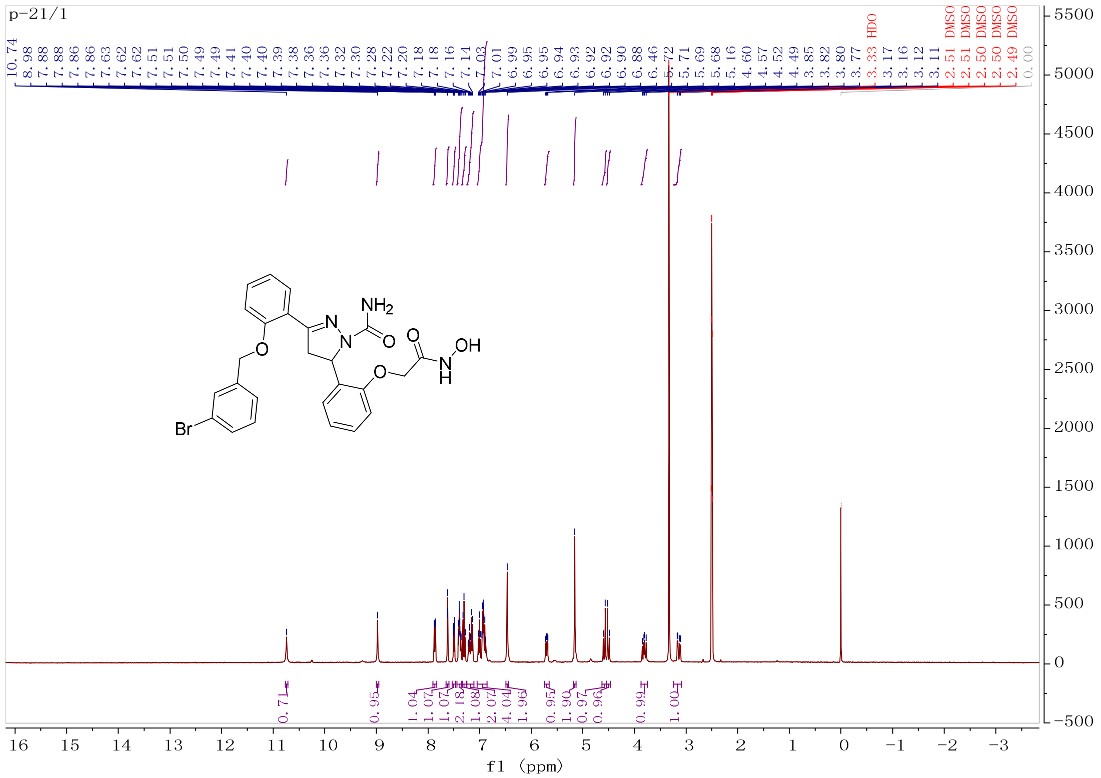

^{1}H NMR spectrum of compound **14ff**

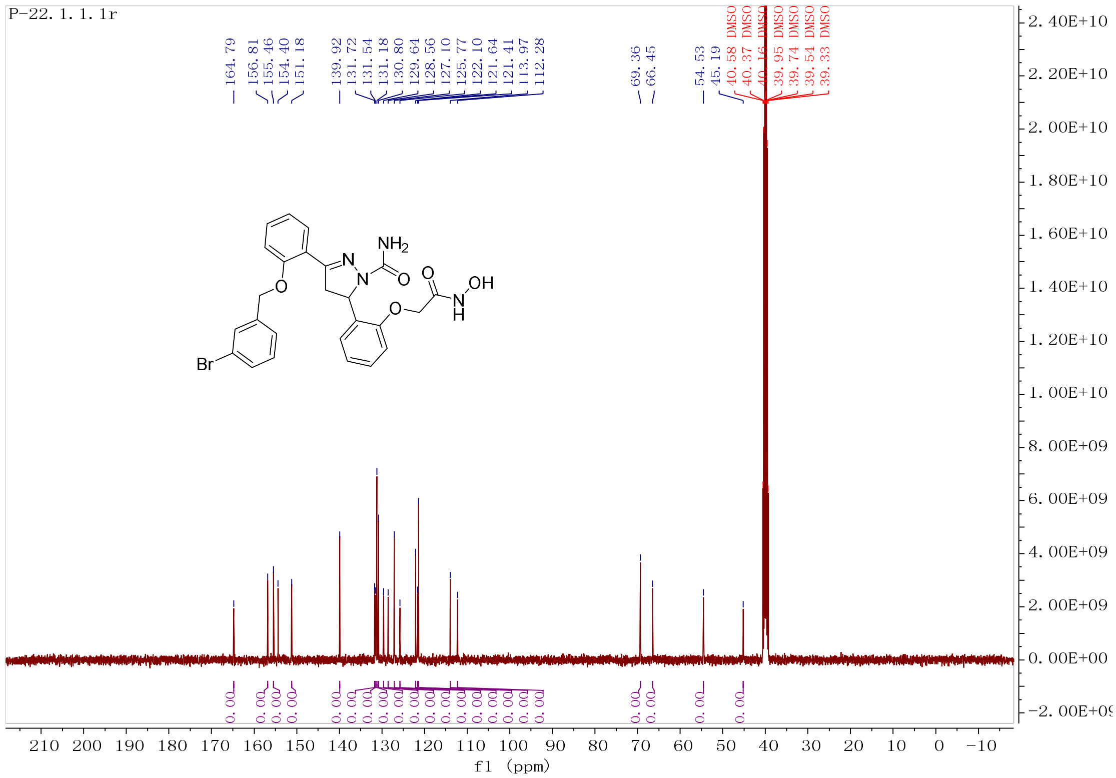

^{13}C NMR spectrum of compound **14ff**
